# Macrophage-expressed IFN-β Contributes to Apoptotic Alveolar Epithelial Cell Injury in Severe Influenza Virus Pneumonia

**DOI:** 10.1371/journal.ppat.1003188

**Published:** 2013-02-28

**Authors:** Katrin Högner, Thorsten Wolff, Stephan Pleschka, Stephanie Plog, Achim D. Gruber, Ulrich Kalinke, Hans-Dieter Walmrath, Johannes Bodner, Stefan Gattenlöhner, Peter Lewe-Schlosser, Mikhail Matrosovich, Werner Seeger, Juergen Lohmeyer, Susanne Herold

**Affiliations:** 1 Department of Internal Medicine II, University of Giessen Lung Center (UGLC) and German Center for Lung Research (DZL), Giessen, Germany; 2 Division of Influenza/Respiratory Viruses, Robert Koch-Institute, Berlin, Germany; 3 Institute of Medical Virology, Justus-Liebig-University, Giessen, Germany; 4 Department of Veterinary Pathology, Freie Universität Berlin, Berlin, Germany; 5 Institute for Experimental Infection Research, Twincore Centre for Experimental and Clinical Infection Research, Hannover, Germany; 6 Division of Thoracic Surgery, University of Giessen Lung Center (UGLC) and German Center for Lung Research (DZL), Giessen, Germany; 7 Department of Pathology, University of Giessen Lung Center (UGLC) and German Center for Lung Research (DZL), Giessen, Germany; 8 Center for Radiation Therapy, University of Giessen Lung Center (UGLC) and German Center for Lung Research (DZL), Giessen, Germany; 9 Institute of Virology, Philipps-University, Marburg, Germany; Mount Sinai School of Medicine, United States of America

## Abstract

Influenza viruses (IV) cause pneumonia in humans with progression to lung failure and fatal outcome. Dysregulated release of cytokines including type I interferons (IFNs) has been attributed a crucial role in immune-mediated pulmonary injury during severe IV infection. Using *ex vivo* and *in vivo* IV infection models, we demonstrate that alveolar macrophage (AM)-expressed IFN-β significantly contributes to IV-induced alveolar epithelial cell (AEC) injury by autocrine induction of the pro-apoptotic factor TNF-related apoptosis-inducing ligand (TRAIL). Of note, TRAIL was highly upregulated in and released from AM of patients with pandemic H1N1 IV-induced acute lung injury. Elucidating the cell-specific underlying signalling pathways revealed that IV infection induced IFN-β release in AM in a protein kinase R- (PKR-) and NF-κB-dependent way. Bone marrow chimeric mice lacking these signalling mediators in resident and lung-recruited AM and mice subjected to alveolar neutralization of IFN-β and TRAIL displayed reduced alveolar epithelial cell apoptosis and attenuated lung injury during severe IV pneumonia. Together, we demonstrate that macrophage-released type I IFNs, apart from their well-known anti-viral properties, contribute to IV-induced AEC damage and lung injury by autocrine induction of the pro-apoptotic factor TRAIL. Our data suggest that therapeutic targeting of the macrophage IFN-β-TRAIL axis might represent a promising strategy to attenuate IV-induced acute lung injury.

## Author Summary

Acute lung injury induced by influenza virus (IV) infection has been linked to an unbalanced release of pro-inflammatory cytokines including type I interferons (IFN) causing immune-mediated organ damage. Using *ex vivo* and *in vivo* IV infection models, we demonstrate that alveolar macrophage-expressed IFN-β induces alveolar epithelial cell injury by autocrine induction of the pro-apoptotic TNF-related apoptosis-inducing ligand (TRAIL). Elucidating the cell-specific underlying signalling pathways revealed that IV-induced IFN-β release from alveolar macrophages (AM) strictly depended on protein kinase R- (PKR-) and NF-κB-signalling. Autocrine activation via the macrophage type I IFN receptor (IFNAR) resulted in increased expression and release of TRAIL which caused apoptosis of IV-infected and non-infected alveolar epithelial cells and promoted alveolar barrier dysfunction as demonstrated in *ex vivo* co-cultures and in bone marrow chimeric mouse models *in vivo*. Importantly, we found TRAIL highly upregulated in and released from AM of hospitalized patients with pandemic H1N1-induced lung failure. Therapeutic targeting of the macrophage IFN-β-TRAIL axis might therefore represent a promising strategy to attenuate IV-induced acute lung injury.

## Introduction

Influenza viruses (IV) can cause primary viral pneumonia in humans with rapid progression to lung failure and fatal outcome [1]. Histopathologic and clinical features of IV-induced lung injury in humans resemble those of other forms of ARDS (acute respiratory distress syndrome), characterized by apoptotic and necrotic alveolar epithelial cell (AEC) damage, loss of alveolar barrier function and severe hypoxemia [1]–[3]. Intrapulmonary expression of pro-apoptotic ligands such as Fas ligand (FasL) or TRAIL has recently been ascribed a key role in induction of epithelial damage during progression to ARDS [4]–[6]. As soon as the infection spreads from the upper to the lower respiratory tract, AEC and resident AM become primary targets for IV infection [3], [7], [8]. Severe IV pneumonia is characterized by an exaggerated alveolar expression of inflammatory mediators and a dysbalance of pro- and anti-inflammatory cytokines causing pulmonary injury, and AM have been attributed a substantial role herein [9]–[11]. However, the key players and molecular mechanisms of the cellular cross-talk during induction of epithelial injury in severe IV pneumonia and the inflammatory mediators involved are still poorly defined. As therapeutic modulation of these pathways offers the potential advantage of exerting less-selective pressure on viral populations than established antiviral drugs, a detailed understanding of the cell-specific inflammatory responses and subsequently induced pathways of epithelial injury in the context of IV infection is of particular importance.

Type I IFN are rapidly expressed in various myeloid and parenchymal cell types following viral infection of the lung [12], [13]. They engage a unique heterodimeric IFN-α receptor (IFNAR) in an autocrine or paracrine way to induce the transcription of various interferon-stimulated genes such as the double-stranded (ds) RNA-dependent protein kinase R (PKR) via the Jak/STAT pathway to restrict viral spread [14], [15]. Moreover, type I IFN signalling was recently ascribed a role in functional differentiation of myeloid cell subsets during IV infection, including monocytes, CD8^+^ T cells, neutrophils and dendritic cells [16]–[18]. Apart from these well-known anti-viral effects, evidence also hints at pathogenic roles for type I IFNs during viral infection. Plenty of pro-inflammatory cytokines and chemokines are induced downstream of IFNAR signalling which may contribute to IV pathogenesis, and disease onset correlates directly with local respiratory production of IFN-α in humans [19]. Thus, type I IFNs may play dual roles in viral clearance and tissue-damaging inflammation.

In the present study we elucidate a previously undefined role of AM-expressed IFN-β in inducing apoptotic AEC damage by expression and release of the pro-apoptotic factor TRAIL upon *ex vivo* and *in vivo* IV infection. Detailed study of the signalling pathways involved in this cellular cross-talk revealed that macrophage IFN-β was released upon IV infection in a PKR- and NF-κB-dependent way and induced autocrine, IFNAR-dependent induction of TRAIL. Macrophage-specific blockade of any of these signalling steps abrogated TRAIL expression in resident and lung-recruited AM, reduced AEC apoptosis and attenuated IV-induced lung injury. These findings highlight type I IFNs as key players in IV-induced pathogenesis and provide new insights into the mechanisms of inflammatory macrophage-epithelial cross-talk in IV-induced lung injury with potential therapeutic implications.

## Results

### IV-induced macrophage-derived IFN-β promotes AEC apoptosis *ex vivo* and *in vivo*

**Figure 1:**
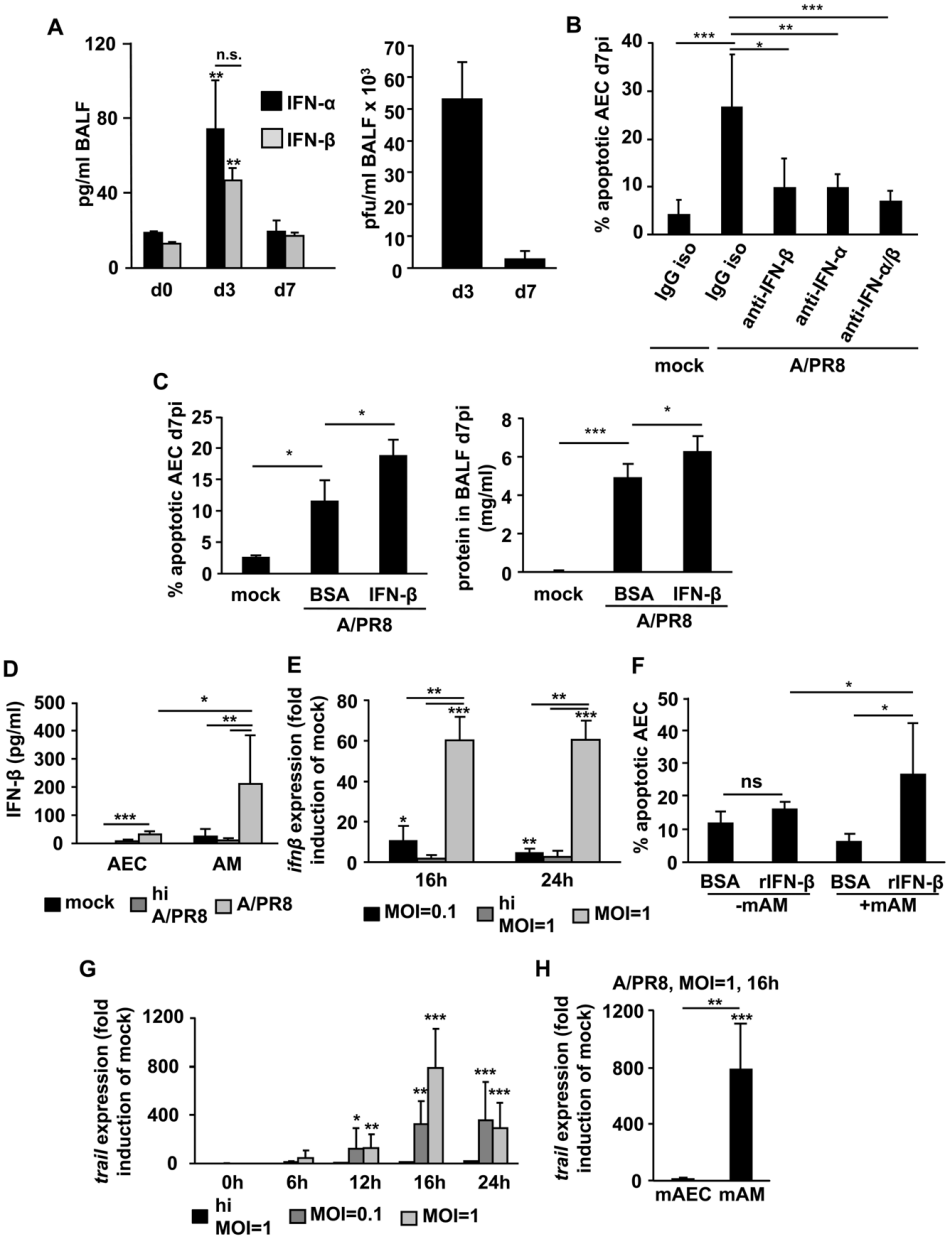
IV-induced IFN-β is mainly macrophage-derived and induces AEC apoptosis *ex vivo* and *in vivo*. (A) C57BL/6 wt mice were infected with 500 pfu A/PR8 and the IFN-α and IFN-β levels (left panel) as well as the amount of infectious virus particles (right panel) were quantified from BALF at indicated time points. (B) AEC apoptosis was quantified in mock or A/PR8 infected mice at d7 pi after intratracheal treatment with IgG isotype Ab, anti IFN-α Ab, anti-IFN-β Ab or both (d5 pi). (C) AEC apoptosis (left panel) and alveolar protein leakage (right panel) were determined at d7 pi after intratracheal treatment with rIFN-β or vehicle (d5 pi). (D) Murine AEC or AM were mock- or A/PR8-infected (live virus or heat-inactivated) *ex vivo* (MOI = 0.1) and IFN-β release was quantified in supernatants at 24 h pi. (E) Murine AM were *ex vivo* infected with live or heat-inactivated A/PR8 at the indicated MOI and IFN-β mRNA expression was quantified at the given times and is depicted as fold induction of mock-infected controls. (F) Uninfected murine AEC were mono- or co-cultured with non-infected AM *ex vivo* in the presence or absence of IFN-β (180 U/ml) for 24 h, and AEC apoptosis rates were quantified. (G) Murine AM were *ex vivo* infected with live or heat-inactivated A/PR8 at the given MOI and TRAIL mRNA expression was quantified and is depicted as fold induction of mock-infected controls. (H) Murine AEC or AM were *ex vivo* infected A/PR8 at the indicated MOI and TRAIL mRNA expression was quantified at the given time and is depicted as fold induction of mock-infected controls. Bar graphs represent means ± SD of (A) 4 animals/group, (B, C) 5 animals/group or of 4 (D, E, G) and 3 (F, H) independent experiments. * p<0.05; ** p<0.01; ***p<0.001; ctrl, control; mAEC, murine alveolar epithelial cells; hi, heat inactivated; Ab, antibody, mAM, murine AM; MOI, multiplicity of infection; pi, post infection; n.s., not significant; iso, isotype; rIFN-β, recombinant IFN-β.

To evaluate the role of type I IFN in IV-induced AEC damage and lung injury, we first quantified type I IFN levels in bronchoalveolar lavage fluid (BALF) during the time course of A/PR8 infection in wildtype (wt) mice. As shown in Fig. 1A (left), IFN-α and -β were both released into the air spaces in response to A/PR8 infection. Corresponding A/PR8 titers in BALF are provided (Fig. 1A, right). To test whether endogenous type I IFNs contributed to the severe lung injury observed in IV-infected mice we analysed AEC injury after alveolar deposition of a neutralizing anti-IFN antibody (Ab). As demonstrated in Fig. 1B, AEC apoptosis was significantly reduced to similar levels when either IFN-α or IFN-β or both were blocked at d5 pi. In turn, alveolar injury at d7 pi was further increased when recombinant IFN-β was applied to the airways of A/PR8-infected mice at d5 pi, as shown in Fig. 1C (left; AEC apoptosis quantified by AnnexinV binding to AEC; right, alveolar protein leakage). Of note, lung viral loads at d7 pi remained unchanged regardless of whether endogenous IFN-β was blocked or recombinant IFN-β was applied intratracheally at d5 pi (data not shown). It is well established that AEC and AM are primary targets for different IV strains in the alveolar compartment of the lung [20]. To determine the predominant source of IV-induced alveolar IFN-β, we next infected primary murine AEC and AM *ex vivo* with A/PR8 and quantified peak IFN-β levels in the culture supernatants. Of note, both AEC and AM were infected by A/PR8 at different MOIs *ex vivo* as determined by immunolabelling of viral nucleoprotein (Fig. S1A). In contrast to AEC, A/PR8 infection of AM was found to be abortive as we could not detect infectious virions in the supernatants of AM cultures. As demonstrated in Fig. 1D, murine AM nevertheless released significantly higher IFN-β levels within 24 h compared to murine AEC in response to infection with live, but not heat-inactivated A/PR8, and this was similarly found when we compared human AM and AEC (Fig. S1B). IFN-β expression robustly occurred in *ex vivo* IV-infected murine AM in a dose- and IV strain-dependent manner (Fig. 1E, S2A), and likewise in human AM (Fig. S2B). To further dissect the mechanisms by which macrophage IFN-β increased AEC apoptosis, we treated non-infected AEC with IFN-β *ex vivo* and determined AEC apoptosis induction. Interestingly, recombinant IFN-β did not increase apoptotic damage in infected mono-cultured AEC, however, AEC apoptosis was highly increased upon IFN-β treatment when AEC were co-cultured with AM (Fig. 1F). Together, these data indicate that macrophage IFN-β induces apoptotic AEC damage upon IV infection and suggest a second, IFN-β-inducible pro-apoptotic macrophage mediator to be involved.

### Macrophage IFN-β induces expression of the pro-apoptotic factor TRAIL upon IV infection in alveolar macrophages

**Figure 2:**
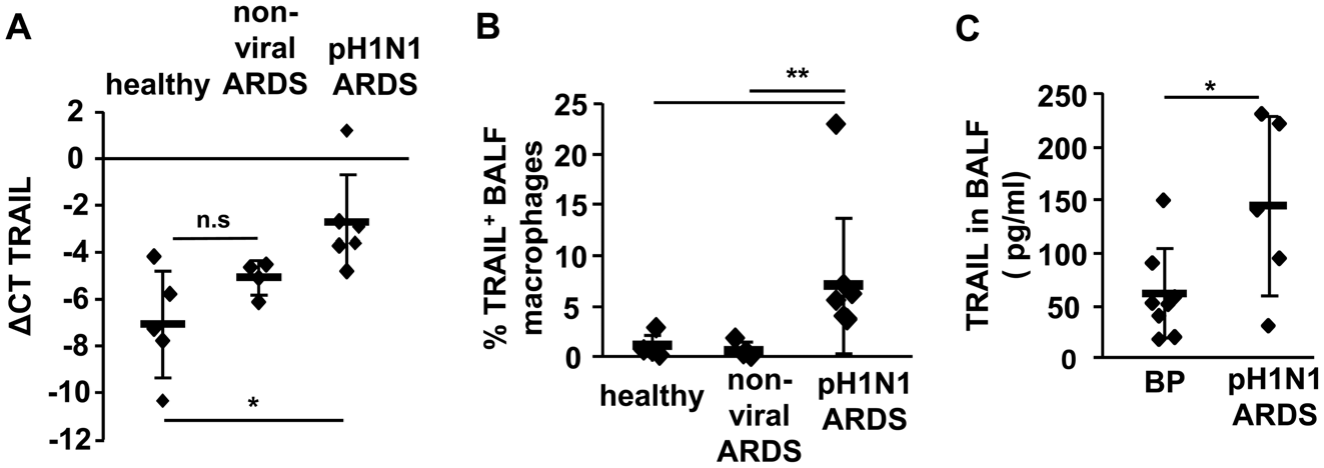
The pro-apoptotic factor TRAIL is upregulated in alveolar macrophages of patients with severe IV pneumonia. AM were isolated from BALF of patients with pH1N1-induced ARDS or non-viral (i.e. bacterial pneumonia- or sepsis-associated) ARDS and TRAIL mRNA (A) and mTRAIL expression (B) were quantified by qRT-PCR and FACS, respectively, and compared to AM of control patients who underwent bronchoscopy due to diagnostic reasons but revealed normal cell numbers and differential counts (‘healthy’ control). C depicts sTRAIL levels in BALF of patients with pH1N1-induced ARDS or with confirmed bacterial pneumonia (BP). The graphs show means ± SD. * p<0.05; ** p<0.01; ***p<0.001; n.s., not significant; mTRAIL, membrane bound TRAIL; sTRAIL, soluble TRAIL.

Several pro-apoptotic factors are known to induce AEC apoptosis during pathogen-related acute lung injury, among which TRAIL and FasL have been reported to be involved [4], [5], [21]. We therefore determined whether TRAIL or FasL were upregulated in murine AM upon *ex vivo* A/PR8 infection. Whereas FasL gene and protein expression were less induced (Fig. S3), TRAIL mRNA was highly upregulated in a dose- and time-dependent manner after A/PR8 infection in AM, with peak TRAIL expression found at 16 h pi at an MOI of 1 (Fig. 1G). Furthermore, TRAIL mRNA was induced after *ex vivo* infection with different IV strains in murine AM (Fig. S2C) and, similarly, in human AM (Fig. S2D). Importantly, TRAIL was increased in alveolar macrophages of hospitalized patients with pandemic H1N1 (pH1N1)-induced ARDS as compared to patients with non-viral ARDS or healthy control individuals, both on mRNA and surface expression level (Fig. 2A, B). Moreover, soluble TRAIL (sTRAIL) levels were significantly higher in BALF of pH1N1-ARDS patients compared to patients suffering from bacterial pneumonia (Fig. 2C), suggesting activation of a TRAIL induction pathway specific for viral infection. Of note, AEC did not increase TRAIL gene expression in response to A/PR8 infection to levels observed in murine AM (Fig. 1H and Fig. S4).

**Figure 3:**
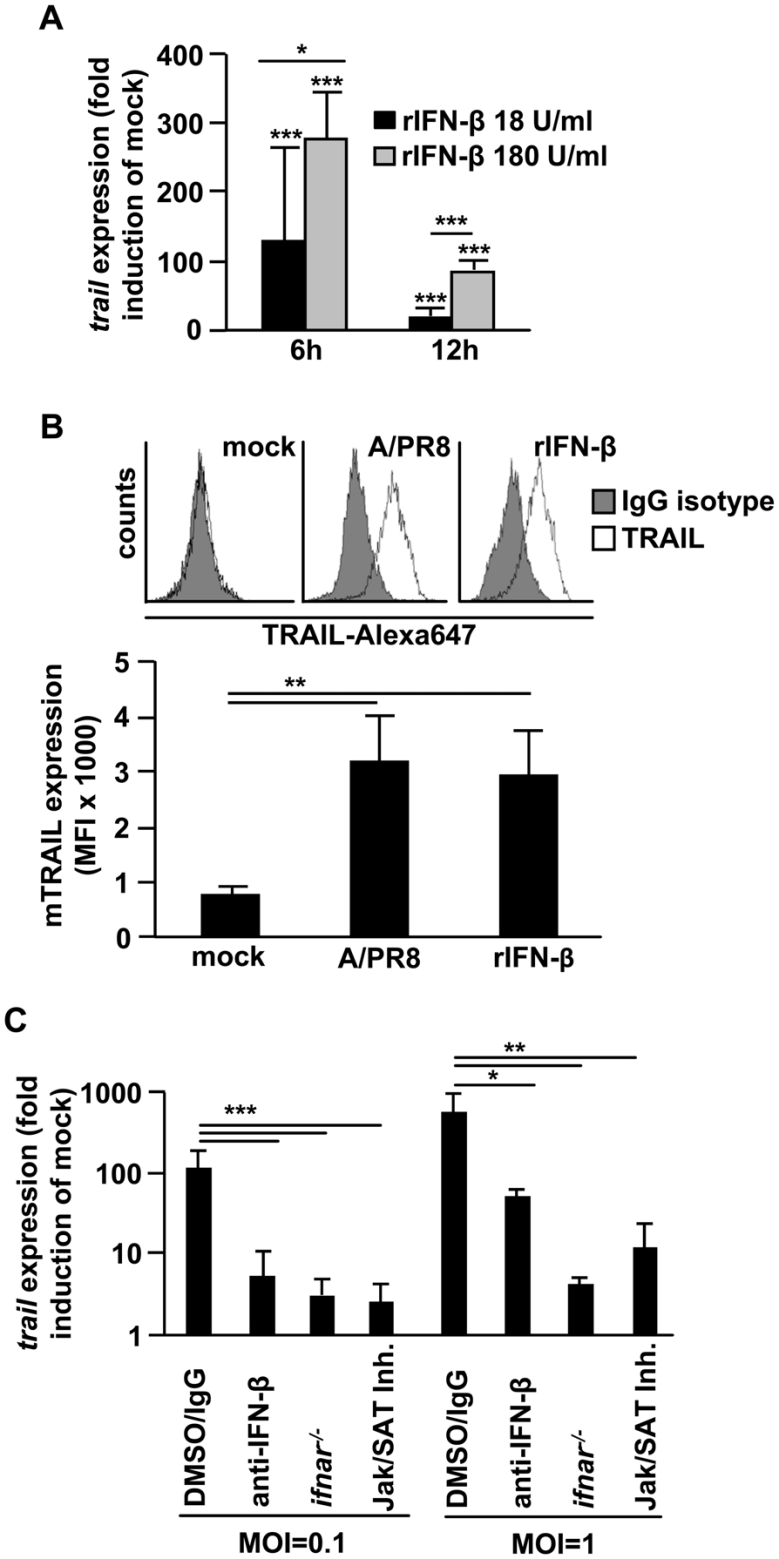
IV-induced macrophage IFN-β upregulates macrophage TRAIL. (A) Murine AM were treated *ex vivo* with rIFN-β at the given concentrations and TRAIL mRNA expression was quantified and is depicted as fold induction of unstimulated controls. (B) Murine AM were A/PR8-infected (MOI = 0.1) or treated with 180 U/ml rIFN-β *ex vivo* in presence of a protease inhibitor cocktail to prevent TRAIL shedding and mTRAIL abundance was analysed by FACS after 24 h. Shown are histograms from a representative experiment (top panel) or mean fluorescence intensities (MFI, bottom panel). (C) Murine wt AM were A/PR8 infected at the given MOI and treated with anti-IFN-β Ab, Jak/STAT inhibitor, or DMSO/isotype IgG Ab as control. Murine *ifnar^−/−^* AM were A/PR8 infected and left untreated. TRAIL mRNA expression was quantified and is depicted as fold induction of mock-infected cells. Bar graphs represent means ± SD of (A) 6; (B) 4 and (C) 5 independent experiments. * p<0.05; ** p<0.01; ***p<0.001; MOI, multiplicity of infection; IgG, IgG isotype control; rIFN-β, recombinant IFN-β; mTRAIL, membrane bound TRAIL; Ab, antibody.

Given the findings that peak TRAIL expression coincided with peak IFN-β expression at 16 h pi in murine AM (Fig. 1E, G) and that TRAIL and IFN-β showed similar IV strain-specific induction patterns in both murine and human AM (Fig. S2), we speculated that macrophage TRAIL expression was dependent on autocrine IFN-β-induced signaling. Indeed, when we stimulated non-infected murine AM with recombinant IFN-β, we detected a time- and dose-dependent increase of TRAIL mRNA (Fig. 3A) and significantly enhanced cell surface TRAIL expression after 24 h of IFN-β stimulation, which was comparable to A/PR8-induced expression (Fig. 3B). Finally, blockade of IFN-β-induced signalling via the type I IFN receptor (IFNAR) nearly abolished A/PR8-induced TRAIL mRNA upregulation in AM regardless of the MOI applied, as demonstrated by either addition of neutralizing anti-IFN-β Ab to the culture medium, use of *ifnar^−/−^* AM, or application of an inhibitor of the IFNAR downstream mediators, Jak and STAT (Fig. 3C). Collectively, these data demonstrate that macrophage-released IFN-β induces expression of the pro-apoptotic factor TRAIL in AM upon IV infection in an autocrine fashion.

### IV-induced IFN-β release and subsequent TRAIL expression depend on activation of PKR and NF-κB in alveolar macrophages

**Figure 4:**
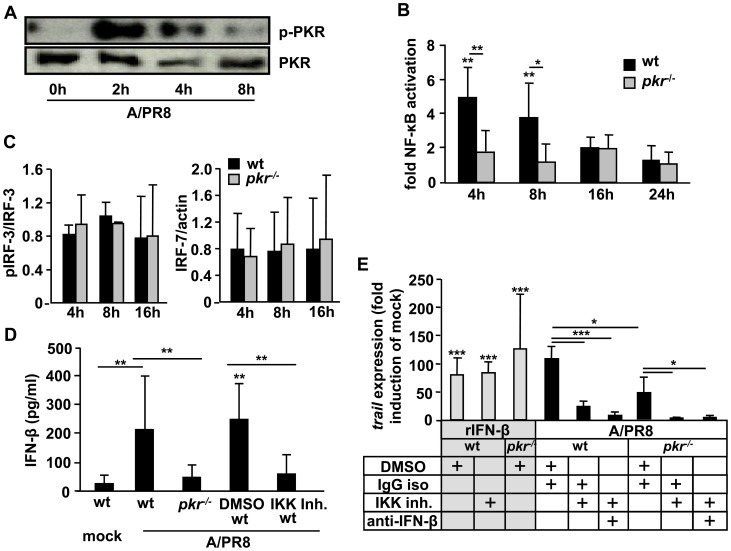
IV-induced IFN-β release and subsequent TRAIL expression depend on activation of PKR and NF-κB in alveolar macrophages. (A) Murine AM were A/PR8 infected *ex vivo* and expression of phosphorylated PKR (p-PKR) and total PKR were assessed by western blot. Wt or *pkr^−/^* ^−^ AM were A/PR8-infected *ex vivo* and NF-κB p65 activation was comparatively analysed by TransAM assay (quantifying p65 binding to a consensus-binding site oligo by a colorimetric method) and depicted as fold activation of mock-infected control (B) or phosphorylated IRF-3 in relation to total IRF-3 protein expression or total IRF-7 expression were determined by western blot at the given time points pi and densitometry data is depicted as relative expression (C). (D) Wt vs. *pkr^−/^* ^−^ AM or IKK inhibitor- vs. DMSO-treated wt AM were A/PR8-infected *ex vivo* and IFN-β concentrations in supernatants were analysed 24 h pi. (E) Wt or *pkr^−/^* ^−^ AM were either A/PR8-infected or stimulated with 180 U/ml rIFN-β, respectively, and additionally treated with IKK inhibitor vs. DMSO or anti-IFN-β vs. isotype Ab and TRAIL mRNA expression was quantified 16 h pi. (A) shows a representative western blot of 3 independent experiments. Bar graphs represent means ± SD of 3 (B, C) or 4 (D, E) independent experiments. * p<0.05; ** p<0.01; ***p<0.005; ctrl, control; iso, isotype; pi, post infection; Ab, antibody.

Several pattern recognition receptors and downstream signalling events were previously shown to be involved in type I IFN induction in IV-infected epithelial cells [22], [23]. However, the cell-specific mechanisms of IV recognition and subsequent IFN-β release in primary AM are far less defined. Given our previous results on protein kinase R (PKR) as important inflammatory signal transducer to NF-κB-mediated transcriptional activity in primary alveolar mouse macrophages [24], we questioned whether the IV-induced type I IFN response in AM similarly depended on PKR, known to directly associate with viral RNA in IV-infected epithelial cells [25]. Indeed, *ex vivo* A/PR8 infection of murine AM resulted in phosphorylation of PKR, most prominent at 2–4 h pi (Fig. 4A). Moreover, the p65 NF-κB subunit was substantially activated at 4–8 h pi in response to A/PR8 infection in wt but not *pkr^−/−^* AM (Fig. 4B). Of note, the infection-triggered activation or expression of transcription factors IRF3 and IRF7, respectively, known to induce IV-dependent type I IFN responses in epithelial cells [23], [26], did not differ in A/PR8-infected wt and *pkr^−/−^* AM (Fig. 4C). To test whether the IV-induced type I IFN response in AM depended on the PKR-NF-κB axis, we quantified IFN-β release from A/PR8-infected wt vs. *pkr^−/−^* AM or in wt AM after NF-κB inhibition. As shown in Fig. 4D, both PKR-deficiency and treatment with a selective IκB kinase (IKK) phosphorylation inhibitor abrogated IFN-β release in A/PR8-infected AM, indicating that IV-induced macrophage IFN-β expression required signal transduction via both PKR and NF-κB. Finally, to address the significance of this signalling pathway for IV-induced TRAIL expression in AM, we stimulated wt or *pkr^−/−^* AM with recombinant IFN-β or infected them with A/PR8 in presence or absence of the IKK inhibitor with or without neutralizing anti-IFN-β Ab. As expected, TRAIL gene expression was significantly induced upon IFN-β stimulation in both wt and *pkr^−/−^* AM, regardless of NF-κB inhibition (Fig. 4E, left gray graphs). However, when AM were A/PR8-infected, TRAIL gene induction depended on presence of PKR and activation of NF-κB. Macrophage IFN-β blockade in addition to NF-κB inhibition did not further decrease TRAIL expression significantly (Fig. 4E, right black graphs). Altogether, these data demonstrate that IV-infection of AM induces a PKR-NF-κB-dependent autocrine IFN-β signaling loop resulting in expression of the pro-apoptotic ligand TRAIL in IV-infected AM.

### The TRAIL receptor DR5 is upregulated in AEC upon IV infection in an IFN-β-independent manner

**Figure 5:**
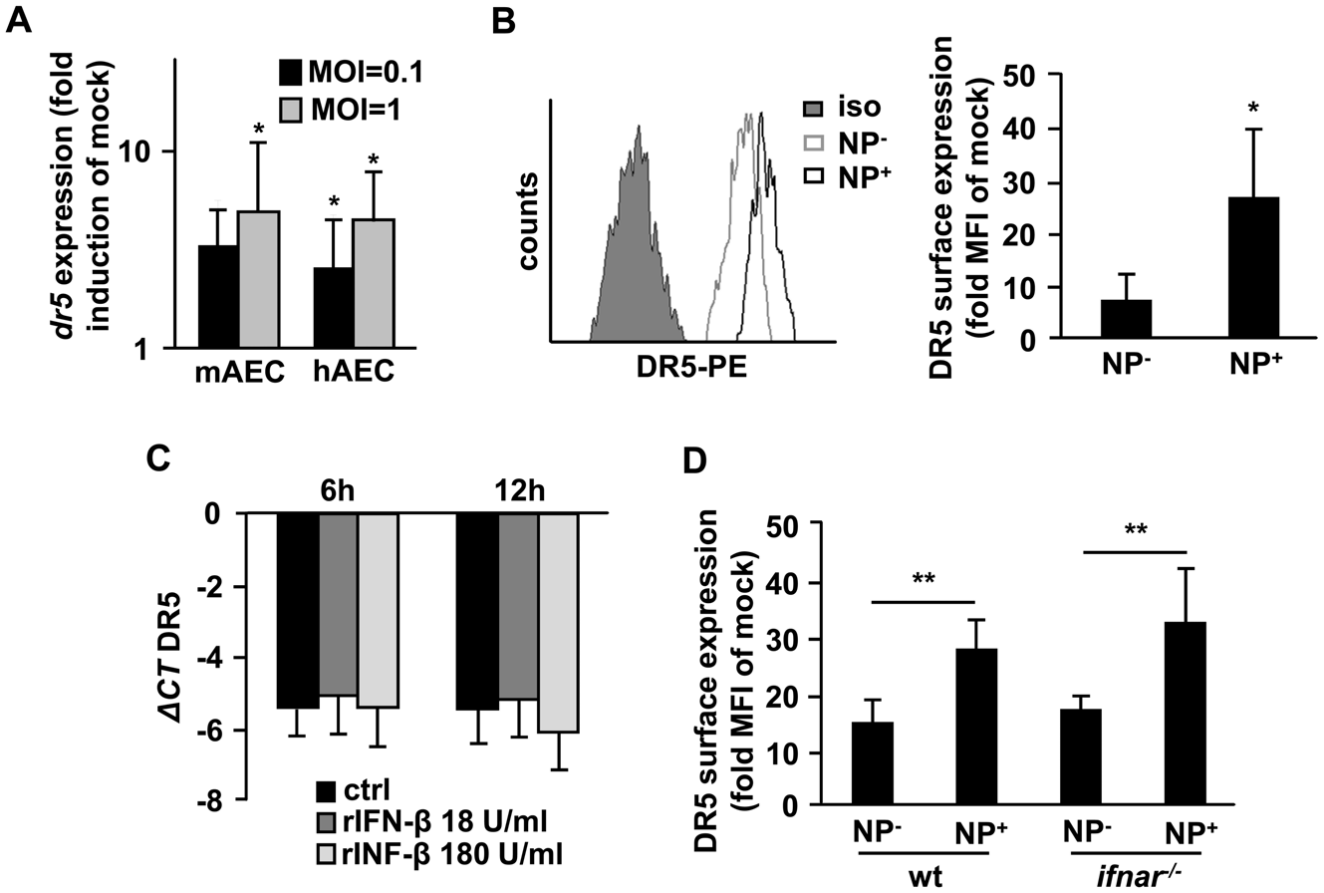
DR5 is upregulated in AEC upon IV infection in an IFN-β-independent way. (A) Primary murine or human AEC were mock- or A/PR8-infected *ex vivo* and DR5 mRNA expression was quantified 48 h pi. (B) Murine AEC were mock- or A/PR8-infected *ex vivo* and surface DR5 abundance in infected (NP^+^) vs. non-infected (NP^−^) AEC from the same culture was analysed by FACS 48 h pi (representative histogram provided in the left panel) and is depicted as fold MFI (mean fluorescence intensity) of mock-infected cultures (right panel). (C) Murine AEC were stimulated with rIFN-β or vehicle-treated (ctrl) and DR5 mRNA expression was quantified after 6 h and 12 h and is depicted as ΔCT values. (D) DR5 surface expression on AEC from A/PR8-infected wt and *ifnar^−/−^* mice was analysed by FACS. Bar graphs represent means ± SD of 4 (A) and 5 (B, C) independent experiments. Bar graphs in D represent means ± SD of animals/group. * p<0,05; ** p<0,01; ***p<0,001; MOI, multiplicity of infection; hAEC, human AEC; mAEC, murine AEC; NP, nucleoprotein; iso, isotype Ab; rIFN-β, recombinant IFN-β.

To determine whether IV-induced AM-expressed IFN-β might additionally promote TRAIL-induced signalling on receptor level, we next quantified AEC TRAIL receptor (DR5, death receptor 5) expression in mono-cultured AEC. As shown in Fig. 5, DR5 was MOI-dependently upregulated in both murine and human AEC upon *ex vivo* A/PR8 infection on gene expression level (Fig. 5A). Of note, A/PR8-infected (IV nucleoprotein^+^, NP^+^) AEC showed significantly increased DR5 surface expression compared to non-infected (NP^−^) AEC within the same culture (Fig. 5B). Interestingly, stimulation of AEC with IFN-β did not impact on DR5 gene expression (Fig. 5C), and DR5 surface expression on AEC did not differ between wt and *ifnar^−/−^* mice in vivo (Fig. 5D), suggesting that macrophage IFN-β induces target cell apoptosis rather by increased macrophage expression of death receptor ligands than by affecting DR5 levels on AEC. Nonetheless, IV-infection sensitizes AEC for TRAIL-mediated killing through IFN-β-independent DR5 upregulation.

### IV-infected alveolar macrophages promote apoptosis in non-infected and IV-infected AEC in a PKR- and type I IFN- dependent way by release of sTRAIL

**Figure 6:**
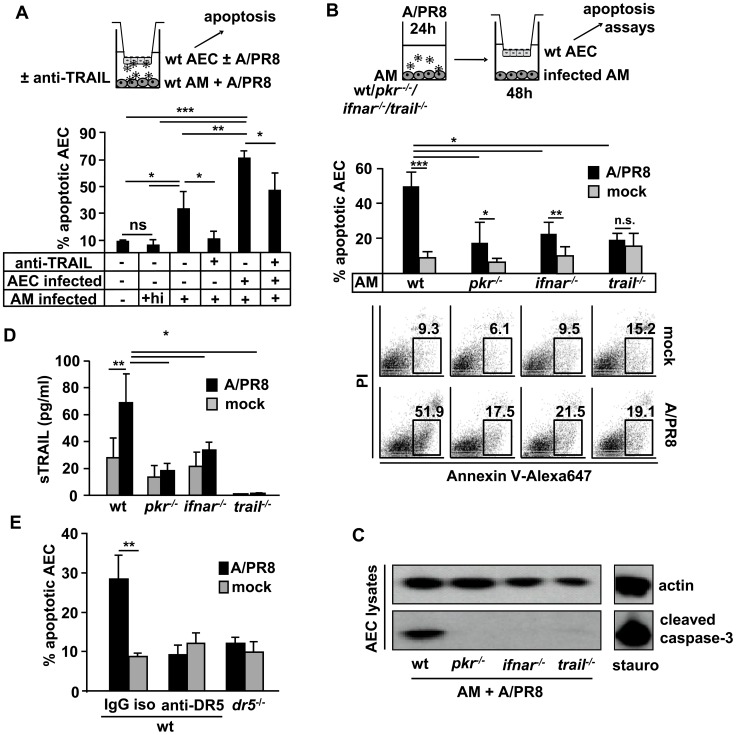
Macrophage TRAIL induces AEC apoptosis in a PKR- and type I IFN-dependent way *ex vivo*. (A) 48 h co-cultured murine AEC (mock- or A/PR8-infected) and AM (A/PR8-infected) were treated with anti-TRAIL or isotype Ab and AEC apoptosis was quantified by flow cytometry (Annexin V staining). (B, C) Mock- or A/PR8-infected wt, *pkr* ^−/−^, *ifnar^−^* ^/−^ or *trail^−/−^* AM (MOI = 0.1) were co-cultured with AEC for 48 h until AEC apoptosis was determined by FACS (representative FACS plots provided in bottom panel) or western blot using an anti-cleaved caspase-3 Ab and lysates of staurosporin-treated AEC as positive control. (D) Wt or gene-deficient AM were mock- or A/PR8-infected (MOI = 0.1) *ex vivo* and TRAIL concentrations were determined at 48 h pi. (E) Mock or A/PR8 infected wt macrophages were co-cultured with wt AEC and a neutralizing anti-DR5 antibody or the isotype control were added to the medium. Additionally, wt mock or A/PR8 infected macrophages were co-cultured with *dr5* ^−/−^ AEC for 48 h and until AEC apoptosis was determined by FACS. (C) shows a representative western blot of 3 independent experiments. Bar graphs show means ± SD of (A) 3, (B, D) 5 and (E) 3 independent experiments. * p<0,05; ** p<0,01; ***p<0,001; AM, alveolar macrophages; AEC, alveolar epithelial cells; hi, heat inactivated; ns, not significant; iso, isotype; sTRAIL, soluble TRAIL.

To address whether epithelial IV-infection was required for macrophage TRAIL-induced apoptosis induction, mock- or A/PR8-infected AECs were co-cultured with infected AM in the presence of anti-TRAIL or control IgG isotype Ab. AEC apoptosis was induced in both mock- and A/PR8-infected co-cultured AEC, however, apoptosis levels in infected AEC, expressing increased levels of DR5 (Fig. 5A and B) exceeded those of mock-infected AEC. Addition of neutralizing anti-TRAIL Ab significantly reduced AEC apoptosis induction in both mock- or A/PR8-infected AEC (Fig. 6A). To address whether the macrophage-specific signalling events mediating IV-induced TRAIL expression indeed promoted AEC apoptosis, we mock- or A/PR8-infected wt, *pkr^−/−^*, *ifnar^−/−^*, or *trail^−/−^* AM for 24 h, co-cultured them with AEC for further 48 h and then analysed the proportion of AnnexinV^+^ AEC. AEC apoptosis rates were not increased upon co-culture with mock-infected wt, *pkr^−/−^*, *ifnar^−/−^*, or *trail^−/−^* AM, compared to uninfected, mono-cultured AEC (8.3±1.8%, data not shown). However, co-culture with infected wt AM strongly induced apoptosis in AEC, and this was significantly reduced when AEC were co-cultured with infected *pkr^−/−^*, *ifnar^−/−^*, or *trail^−/−^* AM (Fig. 6B). Similar data were obtained when expression of cleaved caspase-3 was analysed by western blot in co-cultured AEC (Fig. 6C). Correspondingly, levels of sTRAIL in supernatants were significantly increased upon infection of wt, but not *pkr^−/−^*, *ifnar^−/−^*, or *trail^−/−^* AM (Fig. 6D). Finally, use of blocking DR5 Ab and of AEC derived from DR5-deficient mice in co-cultures revealed that AM-mediated AEC apoptosis induction was dependent on the epithelial TRAIL receptor DR5 (Fig. 6E). Collectively, these data demonstrate that PKR-IFN-β/IFNAR-dependent macrophage TRAIL- induces apoptotic cell death via DR5 in both non-infected and IV-infected AEC *ex vivo*.

### Blockade of autocrine myeloid IFN-β signalling attenuates epithelial injury upon A/PR8 infection *in vivo*

**Figure 7:**
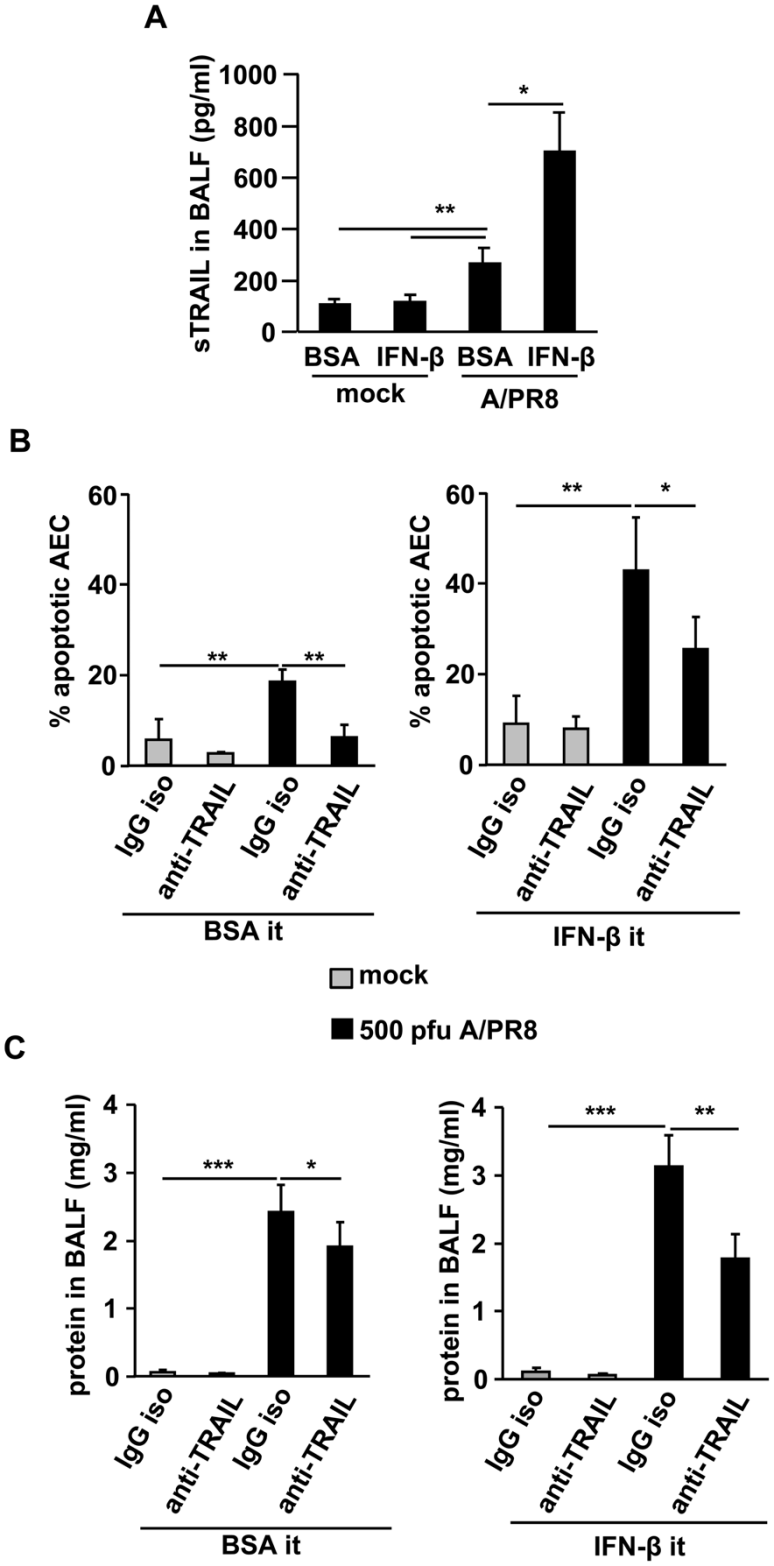
Blockade of IFN-β dependently induced TRAIL attenuates epithelial injury upon IV infection *in vivo*. (A) A/PR8 infected wt mice (500 pfu) were intratracheally treated with rIFN-β (10.000 IU) or vehicle (0.1% BSA in PBS) at d5 pi and sTRAIL was quantified from BALF. (B, C) A/PR8 infected wt mice (500 pfu) were intratracheally treated with rIFN-β or vehicle (0.1% BSA in PBS) and anti-TRAIL Ab or isotype Ab intraperitoneally and AEC apoptosis (B) and alveolar protein leakage (C) were determined at d7 pi. Bar graphs show means ± SD of (A) 6 animals/group and (B, C) 8 animals/group. * p<0,05; ** p<0,01; ***p<0,001. Iso, isotype; sTRAIL, soluble TRAIL; Ab, antibody; pi, post infection.

**Figure 8:**
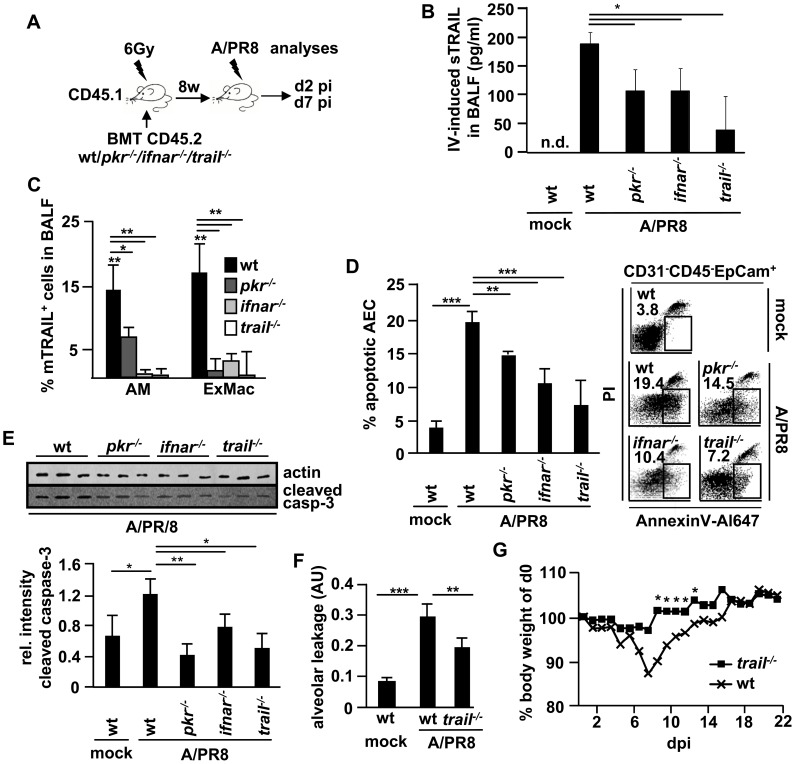
Blockade of autocrine myeloid IFN-β signalling impairs macrophage TRAIL expression and attenuates epithelial injury upon A/PR8 infection *in vivo*. (A) Treatment protocol: CD45.1^+^ wt mice were lethally irradiated (6 Gy) and transplanted 1×10^6^ CD45.2^+^ wt, *pkr^−/−^*, *ifnar^−/−^* or *trail^−/−^* BM cells to generate chimeric mice. 12w later, when >90% of AM were of donor (wt, *pkr^−/−^*, *ifnar^−/−^* or *trail^−/−^*) phenotype, chimeric mice were mock- or A/PR8-infected and subjected to analyses at 2 d or 7 d pi. (B, C) depict IV-induced sTRAIL concentrations in BALF (B) and proportions of mTRAIL-expressing AM and ExMac (exudate macrophages) in BALF of chimeric mice at d2 pi (C). (D, E) AEC apoptosis was quantified in mock- or A/PR8-infected chimeric mice at d7 pi by FACS (D, depicted as Annexin V^+^ proportion of CD31^−^CD45^−^EpCam^+^ lung cells, left panel; representative FACS plots, right panel) or by western blot using lysates of AEC isolated from mock- or A/PR8-infected chimeric mice and a cleaved caspase-3-specific Ab (E, top panel, western blot of 3 independent experiments; bottom panel, quantification of western blot data by densitometry). (F) Alveolar albumin leakage was analysed in mock- or A/PR8-infected wt and *trail^−/^* ^−^ chimeric mice at d7 pi by intravenous injection of FITC-labelled albumin and is depicted as ratio of serum and BALF FITC-fluorescence in arbitrary units (AU). (G) Body weight of wt and *trail^−/^* ^−^ chimeric mice was determined post A/PR8 infection (350 pfu/∼30%LD50). Bar graphs show means ± SD of (B, C, D, E, F) 5 animals/group and (G) 8 animals/group. * p<0,05; ** p<0,01; ***p<0,001; n.d.; not determined; BMT, bone marrow transplantation; dpi, days post infection; pi, post infection; sTRAIL, soluble TRAIL; mTRAIL, membrane bound TRAIL; Ab, antibody.

To investigate whether IFN-β induced pro-apoptotic AM-AEC cross-talk operates also during *in vivo* IV infection, A/PR8-infected wt mice were intratracheally treated with IFN-β at d5 pi. As shown in Fig. 7A, sTRAIL concentrations in BALF at d7 pi were significantly increased compared to vehicle-treated or mock-infected mice. IFN-β-induced (both endogenous and exogenously applied IFN-β) AEC apoptosis and alveolar protein leakage were attenuated upon intratracheal treatment with an anti-TRAIL Ab (Fig. 7B, C). To address the role of PKR activation and subsequent type I IFN release in macrophage TRAIL-induced epithelial cell damage, we next created bone marrow chimeric mice of wt recipient phenotype which were transplanted wt, *pkr^−/−^*, *ifnar^−/−^*, or *trail^−/−^* BM cells. Chimeric mice displayed >90% of circulating and tissue-resident macrophages of donor phenotype at 12w post BMT (Fig. S5), and were subjected to A/PR8-infection as outlined in Fig. 8A. Quantification of sTRAIL in BALF revealed induction in A/PR8-infected wt BM transplanted mice, whereas sTRAIL concentrations were significantly reduced in infected chimeric mice with *pkr^−/−^*, *ifnar^−/−^* or *trail^−/−^* AM (Fig. 8B). Likewise, the proportions of mTRAIL expressing alveolar resident (AM) and recruited exudate (ExMac) macrophages ranged at ∼15% in wt BM transplanted mice, and were widely reduced when resident or exudate macrophages were PKR-, or IFNAR-deficient (Fig. 8C, flow cytometric gating strategies for AM and ExMac depicted in Fig. S6). Notably, mTRAIL was not significantly expressed on macrophages of mock-infected mice or on further myeloid lung-recruited leukocyte populations in BALF (neutrophils; data not shown) or lung homogenates (CD11b^+^ dendritic cells, CD103^+^ dendritic cells; data not shown) in the different groups of chimeric mice after A/PR8 infection. Correspondingly, the fractions of apoptotic AEC, increased in A/PR8-infected compared to mock-infected wt BM transplanted mice, were reduced in mice transplanted *pkr^−/−^*, *ifnar^−/−^* or *trail^−/−^* BM, as demonstrated by flow cytometric quantification of AnnexinV^+^ AEC or levels of cleaved caspase-3 in AEC isolated from mock- or A/PR8-infected chimeric mice (Fig. 8D, E, respectively). Minor differences in between the AnnexinV- and cleaved caspase 3-based AEC apoptosis quantification might result from the analyses of different signalling events within the apoptotic cascade. Of note, viral clearance was not affected in mice lacking leukocyte TRAIL or IFNAR, however, PKR expression in leukocytes was required for effective antiviral host defense as demonstrated by quantification of A/PR8 titers in BALF at d7 pi (Fig. S7). Finally, macrophage TRAIL deficiency was associated with attenuated IV-induced lung injury and reduced morbidity as shown by alveolar albumin leakage at d7 pi (Fig. 8F) and body weight loss in the time course pi (Fig. 8G). Together, these data reveal that TRAIL expression is induced in a PKR- and type I IFN-dependent way in AM upon A/PR8 infection *in vivo* which significantly contributes to IV-induced lung injury.

## Discussion

**Figure 9:**
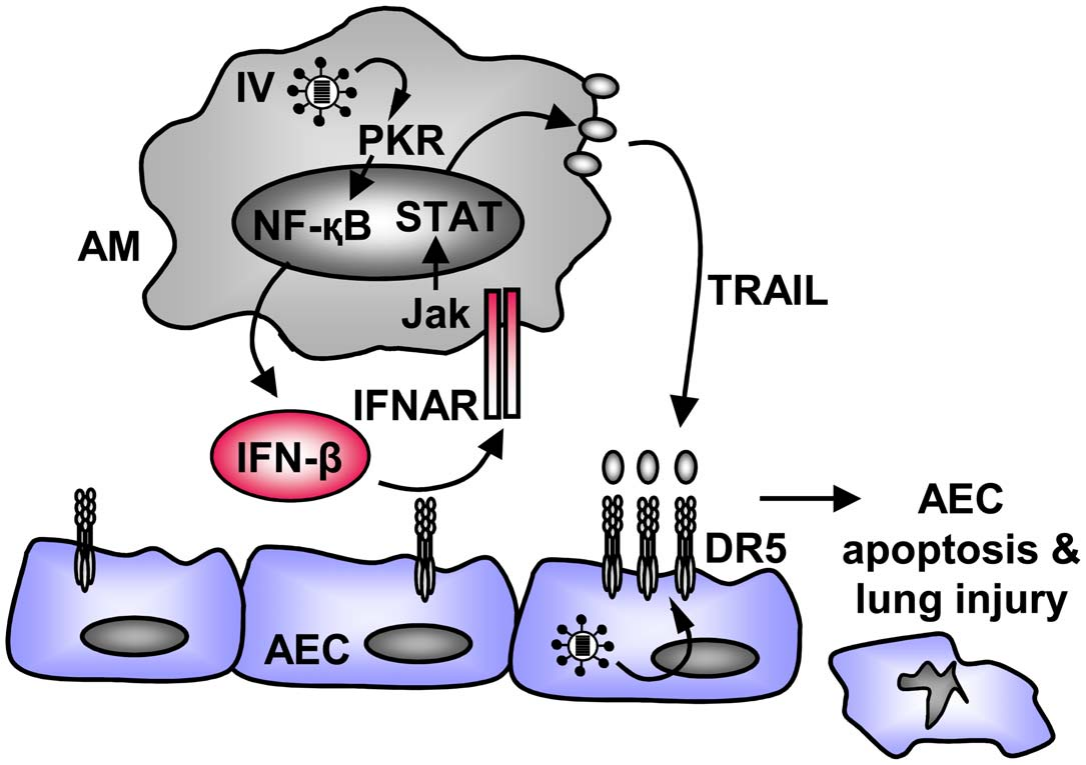
Proposed model of IFN-β mediated pro-apoptotic AM-AEC cross-talk in IV-induced lung injury. IFN-β is released from IV-infected AM in a PKR- and NF-κB-dependent way and induces expression and release of macrophage TRAIL via autocrine IFNAR activation. Macrophage TRAIL induces AEC apoptosis via its receptor DR5 which is constitutively expressed on non-infected and upregulated on IV-infected AEC. Through this signalling cascade, IFN-β significantly contributes to AEC damage and lung injury during severe IV pneumonia. IV, influenza virus.

IV pneumonia is characterized by infection of distal lung epithelial cells and resident macrophages and frequently progresses to acute lung injury/ARDS with poor outcome. Rapid onset of effective host defense strategies is therefore crucial for recovery from IV-induced lung damage. On the other side, an exaggerated inflammatory response will add substantial tissue injury to virus-induced cytopathic damage. This study elucidates a previously unrecognized pathway of cytokine-mediated epithelial injury and highlights the IV-infected alveolar macrophage as key player in this scenario. We demonstrate that IFN-β, induced in IV-infected AM through a PKR-NF-κB-dependent pathway, induces expression and release of the pro-apoptotic ligand TRAIL by autocrine IFNAR activation, and thereby significantly contributes to IV-induced apoptotic tissue injury (Fig. 9).

Although it becomes increasingly evident that influenza viral 5′-triphosphate-linked RNA in epithelial cells is primarily recognized by RIG-I (RNA helicases retinoic acid inducible gene-I) [22], the pathways of IV-dependent type I IFN induction in primary AM have not been addressed in this respect. Given that RIG-I-like helicases signal through the downstream adapter MAVS (mitochondrial antiviral signaling, also called IPS-1) in epithelial IV infection, which mediates IRF3/7-dependent production of type I IFNs [23], we analysed whether these transcriptional effectors were required for IFN-β production in primary AM. However, we did not detect increased expression or activation of IRF7 or IRF3, respectively, upon A/PR8 infection in wt or *pkr^−/−^* AM. In fact, our data for the first time demonstrate that, in AM, PKR signalling and subsequent transcriptional activity of NF-κB are indispensable for type I IFN release upon IV infection, suggesting a macrophage-specific way of signal transduction and adding to a previous study highlighting NF-κB as key regulator of the cellular type I IFN response towards a highly pathogenic IV [27]. The importance of macrophage PKR in the context of IV infection is highlighted by the fact that myeloid PKR-deficiency results in ineffective viral clearance at d7 pi (Fig. S7). The double-stranded (ds) RNA-dependent kinase PKR is a well-characterized component of the antiviral response present in non-stimulated cells at basal levels, and further upregulated by type I IFN [15], [28]. PKR is activated by autophosphorylation upon binding to viral RNP (ribonucleoprotein) complexes and phosphorylates the protein synthesis eukaryotic initiation factor 2a (eIF-2a), resulting in rapid inhibition of translation to limit virion production [25], [29]. More recently, PKR was shown to control transcriptional activation of the NF-κB pathway, independently of its kinase activity, in murine AM and other cells [24], [30]. Whether binding of viral RNPs is required for NF-κB activation or whether PKR rather transduces IV-induced upstream signals in AM is under our current investigation.

Type I IFNs are critical components of the host's innate antiviral response [31], and compounds that trigger this response are clinically in use to treat different viral infections. Accumulating evidence suggests that engagement of the type I IFN response prior to infection may be a therapeutic strategy to control IV infection in different animal models [32], [33]. Furthermore, mice genetically deficient in type I IFN signalling had inefficient IV clearance [16], [34], and *ifnar^−/−^* mice displayed a 50% increased mortality rate compared to wt litters in response to A/PR8 infection (data not shown). Together with our data obtained from the *ifnar^−/−^* BM chimeric mouse model, these findings suggests a minor role of myeloid as opposed to lung parenchymal IFNAR signalling in antiviral host defense, as *ifnar^−/−^* chimeric mice clear IV to a similar extent as control mice (Fig. S7). Our data reveal that AM are a substantial source of IFN-β in the IV-infected lung. Neutralization of alveolar IFN-β and/or -α at d5 pi attenuated AEC injury at d7 pi suggesting that blockade of one of the type I IFNs at the IFNAR receptor is sufficient to strongly reduce AEC apoptosis. Concomitantly, alveolar deposition of recombinant IFN-β at d5 pi increased apoptotic AEC injury in IV-infected lungs, whereas IFN-β pretreatment of mice 24 h prior to infection significantly reduced viral spread and concomitant alveolar protein leakage (data not shown). These data add to the aforementioned studies demonstrating potent anti-viral effects of IFN-β applied before or early in the infection course and suggest that a prolonged or exaggerated type I IFN response at later stages, when virus is virtually cleared from the lungs, might be an important amplifier of a detrimental inflammatory response in IV pneumonia.

A major determinant of this interferon-induced detrimental host response was found to be the potent apoptosis-inducing ligand TRAIL. TRAIL is a well-characterized component of the host's anti-tumour immune response which was thought to exert its pro-apoptotic effects only towards malignant cells [35]. Only recently, we and others demonstrated a role of TRAIL-mediated apoptosis in epithelial injury upon viral pneumonia in mice and humans [5], [6]. However the cell-specific signalling events which regulate TRAIL expression in AM remained elusive. Previous reports indicated that IFN-β-stimulated dendritic cells engage TRAIL to mediate tumour cell killing [36] and suggested TRAIL expression in peripheral blood mononuclear cells to be IFN-dependent [37]. In line with these studies, our data reveal that TRAIL gene expression is induced by interferon via autocrine IFNAR signalling in AM upon *ex vivo* and *in vivo* IV infection. Importantly, IV strains of different pathogenicity induced TRAIL gene expression in murine AM to different extents. The mouse-adapted A/PR8, known to be highly pathogenic in mice, caused an ∼800-fold, and the highly pathogenic avian H5N1 IV A/Thailand/KAN-1/04, which causes severe pneumonia in mice [38], induced an ∼200-fold peak increase in TRAIL expression, whereas infection with the lower pathogenic ×31 stimulated by only ∼8-fold. Likewise, patterns of TRAIL mRNA induction resembled those of IFN-β gene expression. These findings suggest a relation between the extent of TRAIL-mediated apoptotic AEC injury and the pathogenic potential of the respective IV strain. Thus, induction of the type I IFN-TRAIL axis may be considered as novel determinant of IV pathogenicity. Of note, given our findings that AM show various levels of permisseveness for different IV strains (abortive infection of H1N1 IV, high replication rates of highly pathogenic IV, not shown), the extent by which this pathway contributes to lung injury upon infection with IV other than A/PR8 remains to be defined. TRAIL is expressed in a variety of immune cells such as T cells, NK cells, resident tissue macrophages, dendritic cells and circulating monocytes [39]. Previous studies demonstrated that antibody-mediated inhibition of TRAIL signalling resulted in reduced viral clearance in a mouse model of IV pneumonia [40], and that CD8^+^ T cells utilize TRAIL to kill IV-infected alveolar epithelial cells *in vivo* [41], suggesting IV-induced TRAIL as important executor of cytotoxic T lymphocyte (CTL) responses. Although our data obtained from the BM chimeric mouse model suggest a minor role of leukocyte-expressed TRAIL in IV clearance, as mice transplanted *trail^−/−^* BM completely cleared IV at d7 pi (Fig. S7), our findings do not exclude that, apart from FasL or Perforin, antigen-specific CTLs engage TRAIL to specifically kill infected IV target cells. In contrast, macrophage TRAIL-mediated epithelial cell killing was not restricted to IV-infected, DR5 high-expressing AEC, but as well affected non-infected AEC with low level DR5 surface expression (Fig. 5, 6). This suggests that TRAIL released from AM in high amounts upon type I IFN stimulation causes substantial collateral damage to the (uninfected) epithelial barrier, especially when macrophages are present in the airspaces in high numbers, as observed during IV pneumonia [6].

In conclusion, our data reveal a novel role of type I IFNs in induction of apoptotic alveolar epithelial injury and highlight the IV-infected macrophage as central player. Identification of the signalling steps involved in this IFN-β-dependent injurious macrophage-epithelial cross-talk provides new potential targets for therapeutic strategies to attenuate lung injury without compromising the anti-viral immune response during severe IV pneumonia.

## Materials and Methods

### Ethics statement

All animal experiments were conducted according to the legal regulations of the German Animal Welfare Act (Tierschutzgesetz) and approved by the regional authorities of the State of Hesse (Regierungspräsidium Giessen; reference numbers 64/2007, 09/2009, 39/2011). All experiments were performed under ketamine/xylazine anesthesia, and all efforts were made to minimize suffering of infected animals. Human lung tissue was obtained from patients who underwent lobectomy after informed written consent (Departments of Pathology and Surgery, Justus-Liebig-University, Giessen). BALF samples derived from the Universities of Giessen and Marburg (UGMLC) biobank. Use of human lung tissue and BALF samples was approved by the University of Giessen Ethics Committee.

### Mice

C57BL/6 wt mice were purchased from Charles River Laboratories. *trail^−/−^* mice [35] were provided by Amgen Inc. (Thousand Oaks, CA, USA). *pkr^−^* ^/−^ [42] were a kind gift from J. Pavlovic (Institute of Medical Virology, University of Zurich, Switzerland). U. Kalinke provided *ifnar^−/−^* mice [43] that under sponsorship of the Paul-Ehrlich-Institut, Langen, Germany, had been backcrossed to B6N20. *Dr5* ^−/−^ mice were a kind gift from T. Mak (Campbell Family Institute for Breast Cancer Research, Department of Medical Biophysics, University of Toronto, Canada) [44]. B6.SJL-*Ptprc^a^* mice expressing the CD45.1 alloantigen (Ly5.1 PTP) on circulating leukocytes (C57BL/6 genetic background) were obtained from The Jackson Laboratory. Mice were bred under specific pathogen-free conditions.

### Human specimen

Human lung tissue was obtained from lobectomy specimen distal from tumors. BALF material derived from the Universities of Giessen and Marburg Lung Center (UGMLC) biobank. BALF samples from patients who were subjected to bronchoscopy for diagnostic purposes but displayed normal BALF cellularity and differential leukocyte counts (i.e. >90% resident alveolar macrophages) were used for isolation of resident alveolar macrophages for *in vitro* infection experiments or as control samples. pH1N1-BALF samples were collected from patients admitted to the Intensive Care Unit of the Department of Internal Medicine II, UGLC between Dec. 2009 and Jan. 2011 with RT-PCR-confirmed (BALF) pandemic H1N1 infection (bacterial infection was excluded in BALF material). All patients (n = 6, age 42±15 y) required mechanical ventilation due to ALI/ARDS and were subjected to BAL for diagnostic reasons on the day of admission. BALF material from patients who were mechanically ventilated due to non-viral (i.e. bacterial pneumonia- or sepsis-induced) ARDS and samples from patients who were subjected to BAL for diagnostic reasons at the day of admission and revealed bacterial pneumonia (n = 8) were additionally included.

### Reagents

The following anti-mouse mAbs/secondary reagents were used for flow cytometry and immunofluorescence: CD31-FITC (BD Pharmingen, Heidelberg, Germany), CD45-APC-Cy7 (BD Pharmingen, Heidelberg, Germany), EpCam-annexin V-Alexa Fluor 647 (Invitrogen, Karlsruhe, Germany), TRAIL (Biolegend, Uithoorn, Netherlands), IgG2a Isotype (Biolegend, Uithoorn, Netherlands), donkey anti-rat IgG Alexa Fluor 488 (Invitrogen, Karlsruhe, Germany), goat anti-rat IgG-PE (Serotec, Düsseldorf, Germany), mouse-anti influenza NP (Meridian life science, Asbach, Germany), anti-mouse DR5 (R&D, Wiesbaden, Germany), goat anti-mouse IgG Alexa Fluor 647 (Invitrogen, Karlsruhe, Germany), goat anti-mouse IgG Alexa Fluor 555 (Invitrogen, Karlsruhe, Germany), donkey anti-rat IgG Alexa Fluor 488 (Invitrogen, Karlsruhe, Germany), CD45.1-FITC (BD Pharmingen, Heidelberg, Germany), CD45.1-PE (BD Pharmingen, Heidelberg, Germany), CD45.2-APC-Cy7 (Biolegend, Uithoorn, Netherlands), SiglecF-PE (BD Pharmingen, Heidelberg, Germany), CD11c-PE-Cy5.5 (Invitrogen, Karlsruhe, Germany), GR1-PE-Cy7 (Biolegend, Uithoorn, Netherlands), CD3ε-APC (Biolegend, Uithoorn, Netherlands), CD11c-APC (Biolegend, Uithoorn, Netherlands), MHCII-FITC (BD Pharmingen, Heidelberg, Germany), CD103-PE-Cy5.5 (Biolegend, Uithoorn, Netherlands), CD11b-PE-Cy7 (Biolegend, Uithoorn, Netherlands), F4/80-APC (Invitrogen, Karlsruhe, Germany), B220-PE-Cy7 (BD Pharmingen, Heidelberg, Germany). Anti-human TRAIL-PE and the corresponding isotype IgG1κ-PE were from BD Pharmingen (Heidelberg, Germany). For western blot analysis the following anti-mouse antibodies were used: Anti-β-Actin (Biolegend, Uithoorn, Netherlands), anti-IRF3 (Santa Cruz, Heidelberg, Germany), anti-phospho IRF3 (Cell Signalling, Frankfurt a.M., Germany), anti-IRF7 (Santa Cruz, Heidelberg, Germany), anti-PKR (Abcam, Cambridge, UK), anti-phospho PKR (Abcam, Cambridge, UK), anti-cleaved Caspase-3 (Cell Signalling, Frankfurt a. M., Germany) and secondary anti-rabbit IgG-HRP antibody (Cell signalling, Frankfurt a. M., Germany). Polyclonal anti-IFN-β Ab (PBL, Herford, Germany) was used for ex *vivo* neutralization at a concentration of 180 IU/ml. For in *vivo* neutralization 10.000 IU/70 µl of anti-IFN-α or anti IFN-β Ab (PBL, Herford, Germany) were intratracheally administered if not otherwise indicated. The inhibitory anti-TRAIL antibody or corresponding IgG control (both Biolegend, Uithoorn, Netherlands) were used *ex vivo* at a concentration of 500 ng/ml and 150 µg were intraperitoneally injected at day 3 and 5 post infection. 10.000 IU/70 µl recombinant murine IFN-β (PBL, Herford, Germany) diluted in 0.1%BSA or 0.1%BSA alone were applied intratracheally or added to the cell culture supernatants at the given concentrations. Recombinant murine TRAIL (R&D, Wiesbaden, Germany, 100 pg/ml) and Staurosporin (Sigma, Deisenhofen, Germany, 2 µM) were used for *in vitro* experiments.

### AEC isolation and culture

Murine AEC were isolated by the method developed by Corti and colleagues [45] as outlined [46]. 2.0×10^5^ AEC/well were seeded in 24 well plates (BD Biosciences, Heidelberg, Germany). For co-culture experiments, 3.0×10^5^AEC were seeded on the bottom side of 8 µm pore size transwells (BD Biosciences, Heidelberg, Germany) [47]. The cells were kept in DMEM supplemented with HEPES, L-glutamine, 10% FCS and antibiotics for 5 d. Human AEC were isolated as described previously [48] and were kept in HAM's F12 medium (Biochrom, Berlin, Germany) supplemented with 10% FCS and antibiotics for 5 d. The purity of murine and human AEC was assessed using anti-CD45, anti-CD326/EpCam and anti-pro-SP-C antibodies (Biolegend, Uithoorn, Netherlands; Millipore, Eschborn, Germany). Cell suspensions with a purity >90% were used for further experiments.

### Alveolar macrophage isolation and culture

Murine resident alveolar macrophages were isolated by bronchoalveolar lavage (BALF) from lungs of wt or gene-deficient mice as described [47], cultivated in RPMI 1640 containing 2% FCS, L-glutamine and antibiotics and were left to adhere for 2 h before further treatment. In selected experiments, protease inhibitor cocktail (1∶400 dilution, Sigma, Deisenhofen, Germany) was added to the culture medium directly after infection or macrophages were treated with Jak/Stat Inhibitor I (1500 nM in DMSO, Calbiochem, Darmstadt, Germany) or Bay 11-7082 (25 µM in DMSO, Calbiochem, Darmstadt, Germany) or DMSO alone was added 1 h prior to and after infection to the cell culture medium. In co-culture experiments 2.5×10^5^ previously infected or mock-infected murine resident alveolar macrophages/well were combined with murine alveolar epithelial cells seeded on the bottom side of transwells and were co-cultivated for 48 h as previously described [47].

Human resident alveolar macrophages were purified from BALF of patients who were subjected to bronchoscopy for diagnostic purposes but revealed no abnormalities in BALF cellularity or differential leukocyte counts in BALF and seeded at a density of 2.5×10^5^ cells/well or 1.25×10^5^cells/well in a 24- or 48-well plate, respectively. Alveolar macrophages from pandH1N1 or control patients (5.0×10^5^ BALF cells/well) were seeded in 12 well plates, kept in RPMI 1640 containing 50% FCS, L-glutamine and antibiotics and left to adhere for 40 min. Cells were washed several times to remove non-adherent lymphocytes and neutrophils. Macrophage purity was assessed using overall morphological criteria, including differences in cell size and shape which was always >90%.

### *Ex vivo* infection

The following IV strains were used for *ex vivo* infection: A/PR/8/34 (H1N1) (A/PR8), A/X-31 (H3N2) (×31), highly pathogenic avian H5N1 virus isolated from a human fatal case A/Thailand/KAN-1/04 (A/Thai) [49], the swine-origin pandemic H1N1 virus A/Hamburg/5/09 (A/Ham) and seasonal H1N1 virus A/Memphis/14/96-M (A/Mem) [50]. Heat inactivation was performed at 56°C for 30 minutes. Cells were infected (MOI = 1, unless otherwise indicated) in a total volume of 75 µl in 48 well plates or 100 µl in 24well plates diluted in PBS^+/+^ supplemented with 0.1% BSA (Sigma, Deisenhofen, Germany) and antibiotics for 1 h. After removal of the inoculum the respective culture medium supplemented with 0.1% BSA (Sigma, Deisenhofen, Germany), 2 µg/ml Trypsin-TPCK (Worthington, Troisdorf, Germany) and antibiotics was added and cells were incubated at 37°C and 5% CO_2_ for 16–24 h. For further processing, cells were trypsinized with Trypsin/EDTA (PAA, Cölbe, Germany) for FACS analyses or lysed for real-time PCR or western blot.

### *In vivo* treatment protocols

Mice were intratracheally inoculated with 350–500 pfu A/PR8 diluted in sterile PBS^−/−^ in a total volume of 70 µl or mock infected with PBS^−/−^ alone. Mice were sacrificed at the indicated time points and blood and BALF were obtained and handled as previously described [6]. BALF cells were counted with a hemocytometer and quantification of leukocyte subsets was performed by differential cell counts of Pappenheim-stained cytocentrifuge preparations using overall morphological criteria, including differences in cell size and shape of nuclei. Alveolar leakage was analyzed by the lung permeability assay by i.v. injection of FITC-labelled albumin (Sigma, Deisenhofen, Germany) and detection of FITC-fluorescence in serum and BALF, as previously described [6] or by detection of total protein concentrations in BALF by a commercially available spectrophotometric assay (BCA assay, Biorad, München, Germany). For analysis of AEC apoptosis, lungs were digested by intratracheal application of Dispase and processed as outlined previously [6].

### Creation of bone marrow chimeric mice

BM cells were isolated under sterile conditions from the tibias and femurs of wt C57BL/6, *pkr* ^−/−^, *ifnar* ^−/−^ or *trail* ^−/−^ donor mice as previously described after total body irradiation (6 Gy) [6]. As controls for bone marrow engraftment, wt C57BL/6 BM cells (expressing the CD45.2 alloantigen) were transplanted into CD45.1 alloantigen-expressing C57BL/6 mice during each transplantation experiment and the proportion of donor CD45.2-expressing leukocyte populations in blood, BALF and lung homogenate were analyzed by flow cytometry. The proportion of donor circulating and lung-resident myeloid cells was regularly >90% as shown by FACS analyses of the proportions of CD45.2^+^ vs. CD45.2^+^ cells. Chimeric mice were housed under specific pathogen free conditions for 12 weeks before PR/8 infection.

### RNA isolation and real time RT-PCR

After cell lysis in RLT buffer (Qiagen, Hilden, Germany), RNA was isolated using RNeasy mini kit (Qiagen, Hilden, Germany) according to the manufacturer's instructions. For cDNA synthesis, reagents and incubation steps were performed as outlined previously [46]. Reactions were performed in a Step one plus Sequence detection system (Applied Biosystems, Foster City, CA) using Sybr green as fluorogenic probe in 25 µl reactions containing 5 µl cDNA sample, Platinum Sybr Green qPCR supermix (Invitrogen, Karlsruhe, Germany) and 45 pmol of forward and reverse primer. The following forward and reverse primers were used: Murine actin forward, 5′-ACCCTAAGGCCAACCGTGGC-3′, reverse 5′-CAGAGGCATACAGGGACAGCA-3′; human Actin forward, 5′-CTGGGAGTGGGTGGAGGC-3′, reverse 5′-TCAACTGGTCtCAAGTCAGTG-3′; murine TRAIL forward, 5′-GAAGACCTCAGAAAGTGGC-3′ and reverse 5′-GACCAGCTCTCCATTCTTA-3′; human TRAIL forward 5′-GAGGTTGCAGTGGTGAGA-3′, reverse 5′-CCCCTGCTGGCAAGTCAA-3′; murine IFN-beta 5′-ACGTCTCCTGGATGAACTCCA-3′ and reverse 5′-CAGTTGAGGACATCTCCCACG-3′; human IFN-β forward, 5′-CAGCAATTTTCAGTGTCAGAAGC-3′, reverse 5′-TCATCCTGTCCTTGAGGCAGT-3′; murine DR5 forward 5′-AAGTGTGTCTCCAAAACGG-3′, and reverse 5′-AATGCACAGAGTTCGCACT-3′; human DR5 forward 5′-GGTTCCAGCAAATGAAGGTG-3′ and reverse 5′-GAGTCAAAGGGCACCAAGTC-3′. Relative gene abundance to housekeeping gene was calculated as a ΔCt value, with ΔCt = Ct _reference_−Ct _target_, and data are presented as ΔCT or fold expression (2^ΔΔCt^) values in between treatment groups.

### Western blot

Cultured cells or AEC isolated from A/PR8 infected mice were lysed in lysis buffer (20 mM Tris-HCL, 150 mM NaCl, 1 mM Na_2_EDTA, 1 mM EGTA, 0.5% NP-40, 2 mM sodium orthovanadate and protease inhibitor cocktail; Roche, Mannheim, Germany) and the protein content was determined using a commercially available spectrophotometric assay (BCA assay, Biorad, München, Germany). Separation of proteins was resolved on a SDS-PAGE and transferred onto Hybond PVDF-membrane (GE healthcare; München, Germany). Membranes were blocked and incubated with the primary and secondary antibodies. The bands were detected using the enhanced chemiluminescent western blotting system (GE healthcare, München, Germany).

### Viral titers

Virus titers in BALF were determined using plaque assay in Madin-Darby canine kidney cells. In brief, duplicate cell monolayers grown in 6-well plates were incubated with 1 ml BALF dilution for 1 h at room temperature and covered with 1.25% Avicel semi-solid overlay medium containing 2 µg/ml Trypsin-TPCK (Worthington, Troisdorf, Germany) for 48 h. Cells were fixed with 4%PFA in PBS, permeabilized with 1%Triton X-100, incubated with diluted primary anti-NP (Meridian Life Science, Asbach, Germany) and secondary HRP-conjugated anti-mouse antibody (Santa Cruz, Heidelberg, Germany) for 45 min, respectively, and stained with True Blue (KPL, Gaithersburg, MD, USA). Plaques were counted using a light microscope (DM 2500, Leica Microsystems, Wetzlar, Germany).

### Flow cytometry

1–5×10^5^ cells were washed in FACS buffer (PBS^−/−^, 7.4% EDTA, 0.5% FCS, pH 7.2) and fixed in 1%PFA/PBS^−/−^ or resuspended in annexin V staining buffer (10 mM HEPES, 140 mM NaCl and 2.5 mM CaCl_2_), incubated with the respective primary and secondary antibodies for 15 min at 4°C, and flow cytometric analysis was performed using a FACSCanto flow cytometer (BD Biosciences, Heidelberg, Germany) and a FACS Diva Software.

### Immunofluorescence

Cells cultured in chamber slides were fixed for 5 min in a mixture of methanol∶acetone (1∶1) and incubated for 30 min in 3% BSA in PBS. The cells were stained for 2 h with the primary and after washing for 2 h with the secondary antibody. After a final washing step the cells were covered with a DAPI-containing mounting medium (Vectashield; Vector Labs, Eching, Germany) and left to dry overnight. Samples were analysed with a Leica DM 2000 light microscope using a Leica digital imaging software (Wetzlar, Germany).

### ELISA and TransAM activity assay

Murine and human IFN-α, IFN-β, TRAIL and FasL levels were analyzed from cell culture supernatants, murine or human BALF or supernatants of homogenized lungs using commercially available ELISA Kits (R&D Systems, Wiesbaden, Germany; PBL, Herford, Germany; USCN, Basel, Switzerland) according to the manufacturer's instructions. Activated NF-κB was quantified using a commercially available TransAM Assay p65 (active motif, La Hulpe, Belgium) according to the manufacturer's instructions. Briefly, the TransAM assay quantifies activated p65 binding to an immobilized consensus-binding site oligo after addition of nuclear extracts. A primary antibody specific for an epitope on the bound and active form of p65 is then added followed by subsequent incubation with secondary antibody and developing solution for colorimetric quantification.

### Statistics

All data are given as mean ± standard deviation. Statistical significance between two groups was estimated using the two-tailed paired or the unpaired student's t-test for paired or unpaired samples, respectively. For comparison of>two groups with each other one-way ANOVA and post-hoc Tukey-HSD was applied. Significancies were calculated with the SPSS for Windows software program. A value of *p*<0.05 was considered as significant.

## Supporting Information

Figure S1 **A/PR8 infection rates of murine AM and AEC** ***ex vivo*** **and IFN-β expression in human alveolar macrophages and epithelial cells upon A/PR8 infection.** (A) Murine AM and AEC were A/PR8 infected with the indicated MOI and the percentage of NP^+^ cells was determined at 8 h, 16 h and 24 h pi. (B) Human AM or AEC were *ex vivo* A/PR8 infected using an MOI = 1 and IFN-β mRNA expression was quantified at the given times and is depicted as fold induction of mock-infected controls. Bar graphs represent means ± SD of 4 (A, B) independent experiments. * p<0.05; ** p<0.01; ***p<0.001. n.d., not detectable; ns, not significant; AM, alveolar macrophages; AEC, alveolar epithelial cells; pi, post infection; NP, nucleoprotein; MOI, multiplicity of infection.
(TIF)

Figure S2 **IFN-β and TRAIL expression are dependent on the influenza virus strain in murine and human alveolar macrophages.** Murine (upper and lower left panel) or human AM (upper and lower right panel) were *ex vivo* infected with the indicated IV and MOI and IFN-β (upper left and right panel) or TRAIL (lower left and right panel) mRNA expression was quantified at the given times and is depicted as fold induction of mock-infected controls. Bar graphs represent means ± SD of (A, C) n = 3 and (B, D) n = 5 independent experiments. * p<0.05; ** p<0.01; ***p<0.001; mAM, murine AM; hAM, human AM; MOI, multiplicity of infection; pH1N1, swine originated pandemic H1N1; sH1N1, seasonal H1N1.
(TIF)

Figure S3 **FasL expression in infected alveolar macrophages.** Murine AM were *ex vivo* infected with A/PR8 with the indicated MOI and FasL mRNA expression was quantified at the given times and is depicted as fold induction of mock-infected controls (A). Murine AM were *ex vivo* infected with A/PR8 with the indicated MOI and sFasL levels were quantified in the cell culture supernatants (B). Bar graphs represent means ± SD of 3 independent experiments. * p<0.05; ** p<0.01; ***p<0.001; MOI, multiplicity of infection, sFasL, soluble Fas Ligand.
(TIF)

Figure S4 **Time course of TRAIL expression in IV-infected AEC.** Murine AEC were *ex vivo* infected with A/PR8 at the indicated MOI and TRAIL mRNA expression was quantified at the given times and is depicted as fold induction of mock-infected controls. Bar graphs represent means ± SD of 3 independent experiments. * p<0.05; ** p<0.01; ***p<0.001; MOI, multiplicity of infection; AEC, alveolar epithelial cells.
(TIF)

Figure S5 **Efficiency of BM reconstitution and exchange of resident AM in chimeric mice.** To control transplantation and resident AM reconstitution efficiency in CD45.1^+^ recipient mice after BMT with CD45.2^+^ BM, the fractions of CD45.2^+^ of total peripheral blood leukocytes (PBL) and of resident AM were quantified by flow cytometry from blood or BALF at 12w post BMT. Bar graphs show means ± SD from 3 independent experiments.
(TIF)

Figure S6 **Gating strategies for detection of resident AM and exudate macrophages (ExMac) from BALF.** Donor leukocytes (CD45.2^+^) were gated on the SSC^high^CD3ε^−^ fraction to exclude lymphocytes. Neutrophils were defined as CD11c^−^GR-1^high^ (PMN). ExMac were defined as CD11c^high^SiglecF^low^GR-1^int^ whereas resident AM were defined as CD11c^high^SiglecF^high^ GR-1^low^.
(TIFF)

Figure S7 **Viral loads in BALF of chimeric wt mice after transplantation of wt,** ***pkr^−/−^*** **,** ***ifnar^−/−^*** **or** ***trail^−/−^*** **BM at d7 pi.** Bar graphs show means of pfu (plaque forming units)×10^3^ ± SD of 5 animals/group. n.s., not significant.
(TIFF)
