# Correction: Macrophage-expressed IFN-β Contributes to Apoptotic Alveolar Epithelial Cell Injury in Severe Influenza Virus Pneumonia

**DOI:** 10.1371/journal.ppat.1005716

**Published:** 2016-06-15

**Authors:** Katrin Högner, Thorsten Wolff, Stephan Pleschka, Stephanie Plog, Achim D. Gruber, Ulrich Kalinke, Hans-Dieter Walmrath, Johannes Bodner, Stefan Gattenlöhner, Peter Lewe-Schlosser, Mikhail Matrosovich, Werner Seeger, Juergen Lohmeyer, Susanne Herold

The authors would like to correct Fig 8D, as errors were introduced in the preparation of the figure for publication. In the right panel of Fig 8D, the ifnar-/- plot is incorrect, a pkr-/- plot was used in place of the correct ifnar-/- plot. The corrected Fig 8, shown here, includes the correct infar-/- plot.

**Fig 8:**
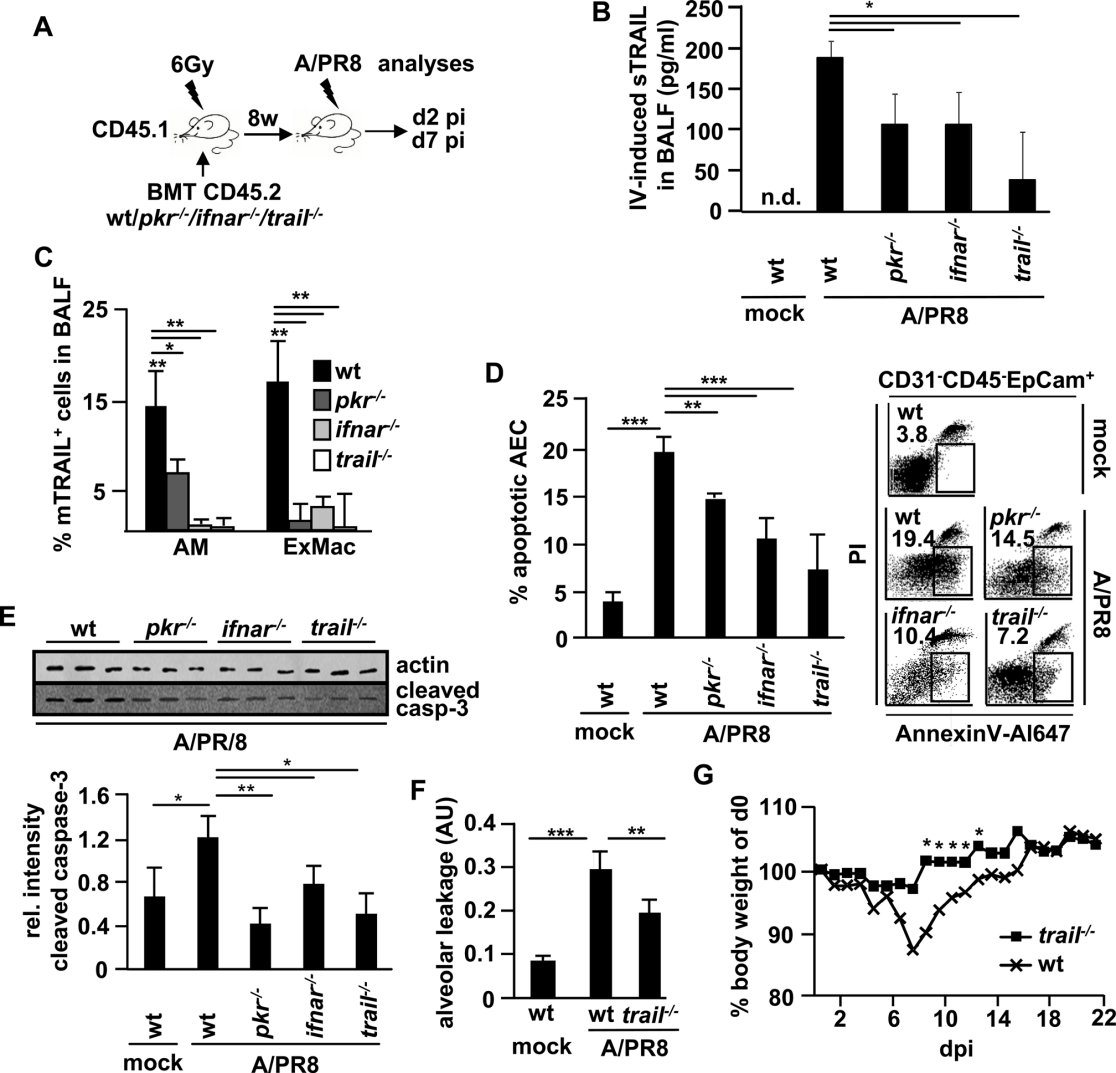
Blockade of autocrine myeloid IFN-β signalling impairs macrophage TRAIL expression and attenuates epithelial injury upon A/PR8 infection *in vivo*. (A) Treatment protocol: CD45.1^+^ wt mice were lethally irradiated (6 Gy) and transplanted 1×10^6^ CD45.2^+^ wt, *pkr* ^*−/−*^, *ifnar* ^*−/−*^ or *trail* ^*−/−*^ BM cells to generate chimeric mice. 12w later, when >90% of AM were of donor (wt, *pkr* ^*−/−*^, *ifnar* ^*−/−*^ or *trail* ^*−/−*^) phenotype, chimeric mice were mock- or A/PR8-infected and subjected to analyses at 2 d or 7 d pi. (B, C) depict IV-induced sTRAIL concentrations in BALF (B) and proportions of mTRAIL-expressing AM and ExMac (exudate macrophages) in BALF of chimeric mice at d2 pi (C). (D, E) AEC apoptosis was quantified in mock- or A/PR8-infected chimeric mice at d7 pi by FACS (D, depicted as Annexin V^+^ proportion of CD31^−^CD45^−^EpCam^+^lung cells, left panel; representative FACS plots, right panel) or by western blot using lysates of AEC isolated from mock- or A/PR8-infected chimeric mice and a cleaved caspase-3-specific Ab (E, top panel, western blot of 3 independent experiments; bottom panel, quantification of western blot data by densitometry). (F) Alveolar albumin leakage was analysed in mock- or A/PR8-infected wt and *trail* ^*−/*−^ chimeric mice at d7 pi by intravenous injection of FITC-labelled albumin and is depicted as ratio of serum and BALF FITC-fluorescence in arbitrary units (AU). (G) Body weight of wt and *trail* ^*−/*−^chimeric mice was determined post A/PR8 infection (350 pfu/∼30%LD50). Bar graphs show means ± SD of (B, C, D, E, F) 5 animals/group and (G) 8 animals/group. * p<0,05; ** p<0,01; ***p<0,001; n.d.; not determined; BMT, bone marrow transplantation; dpi, days post infection; pi, post infection; sTRAIL, soluble TRAIL; mTRAIL, membrane bound TRAIL; Ab, antibody.

The authors confirm that these changes do not alter their findings.

## References

[1] Högner K, Wolff T, Pleschka S, Plog S, Gruber AD, Kalinke U, et al (2013) Macrophage-expressed IFN-β Contributes to Apoptotic Alveolar Epithelial Cell Injury in Severe Influenza Virus Pneumonia. PLoS Pathog 9(2): e1003188 doi:10.1371/journal.ppat.1003188 23468627 10.1371/journal.ppat.1003188 PMC3585175

