# Supplementary figures and images for: Macrophage-expressed IFN-β Contributes to Apoptotic Alveolar Epithelial Cell Injury in Severe Influenza Virus Pneumonia
Source: PLoS Pathog. 2013 Feb 28;9(2):e1003188. doi: 10.1371/journal.ppat.1003188 (PMC3585175; doi:10.1371/journal.ppat.1003188)

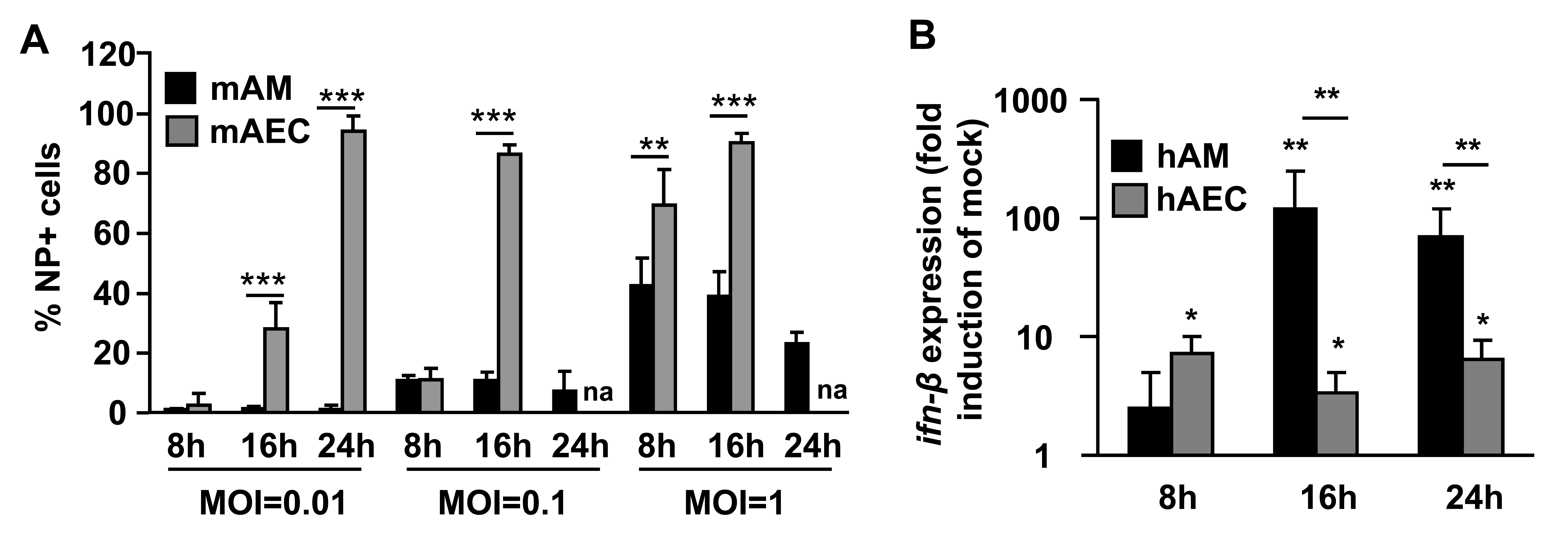

Supplement: Figure S1 — A/PR8 infection rates of murine AM and AEC ex vivo and IFN-β expression in human alveolar macrophages and epithelial cells upon A/PR8 infection. (A) Murine AM and AEC were A/PR8 infected with the indicated MOI and the percentage of NP+ cells was determined at 8 h, 16 h and 24 h pi. (B) Human AM or AEC were ex vivo A/PR8 infected using an MOI = 1 and IFN-β mRNA expression was quantified at the given times and is depicted as fold induction of mock-infected controls. Bar graphs represent means ± SD of 4 (A, B) independent experiments. * p<0.05; ** p<0.01; ***p<0.001. n.d., not detectable; ns, not significant; AM, alveolar macrophages; AEC, alveolar epithelial cells; pi, post infection; NP, nucleoprotein; MOI, multiplicity of infection. (TIF) [file ppat.1003188.s001.tif]

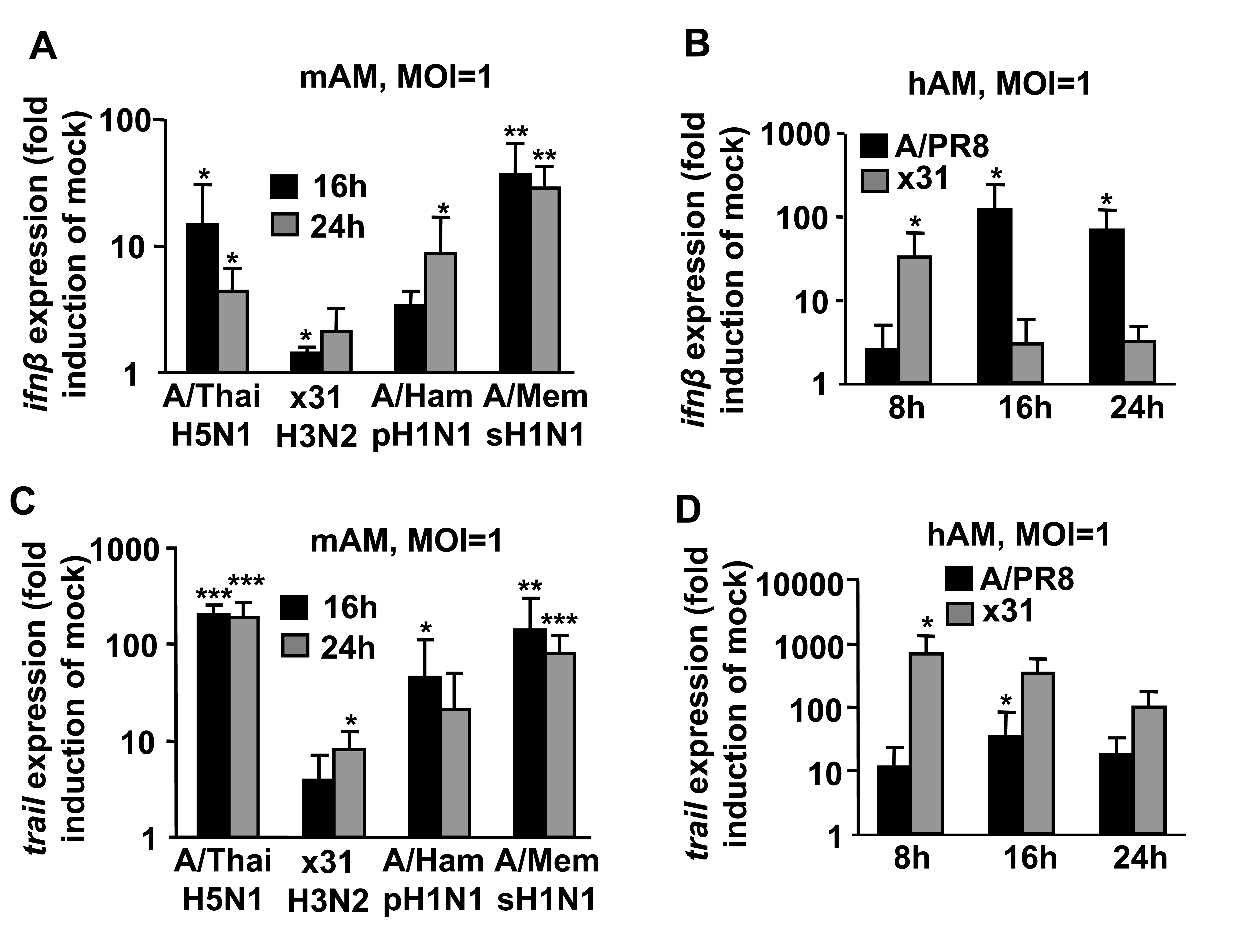

Supplement: Figure S2 — IFN-β and TRAIL expression are dependent on the influenza virus strain in murine and human alveolar macrophages. Murine (upper and lower left panel) or human AM (upper and lower right panel) were ex vivo infected with the indicated IV and MOI and IFN-β (upper left and right panel) or TRAIL (lower left and right panel) mRNA expression was quantified at the given times and is depicted as fold induction of mock-infected controls. Bar graphs represent means ± SD of (A, C) n = 3 and (B, D) n = 5 independent experiments. * p<0.05; ** p<0.01; ***p<0.001; mAM, murine AM; hAM, human AM; MOI, multiplicity of infection; pH1N1, swine originated pandemic H1N1; sH1N1, seasonal H1N1. (TIF) [file ppat.1003188.s002.tif]

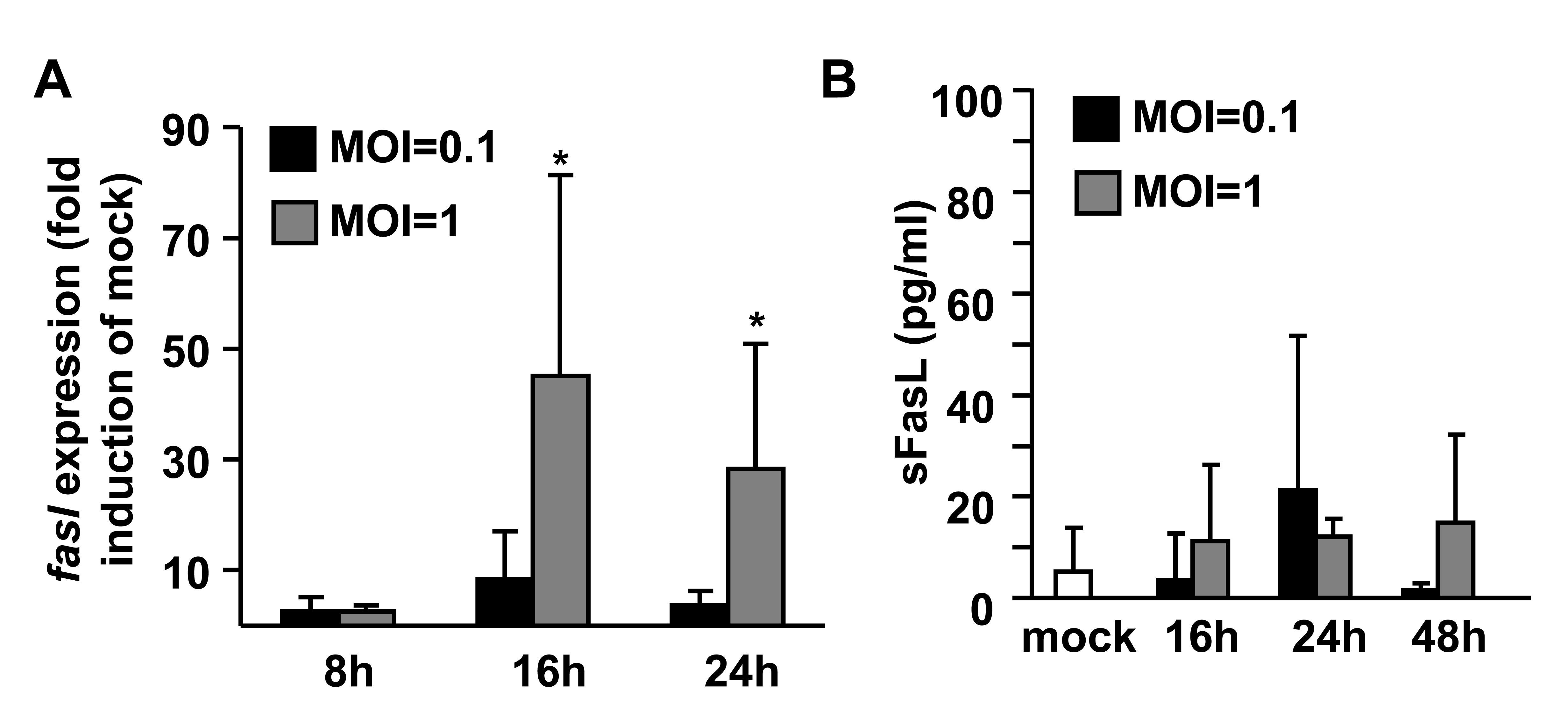

Supplement: Figure S3 — FasL expression in infected alveolar macrophages. Murine AM were ex vivo infected with A/PR8 with the indicated MOI and FasL mRNA expression was quantified at the given times and is depicted as fold induction of mock-infected controls (A). Murine AM were ex vivo infected with A/PR8 with the indicated MOI and sFasL levels were quantified in the cell culture supernatants (B). Bar graphs represent means ± SD of 3 independent experiments. * p<0.05; ** p<0.01; ***p<0.001; MOI, multiplicity of infection, sFasL, soluble Fas Ligand. (TIF) [file ppat.1003188.s003.tif]

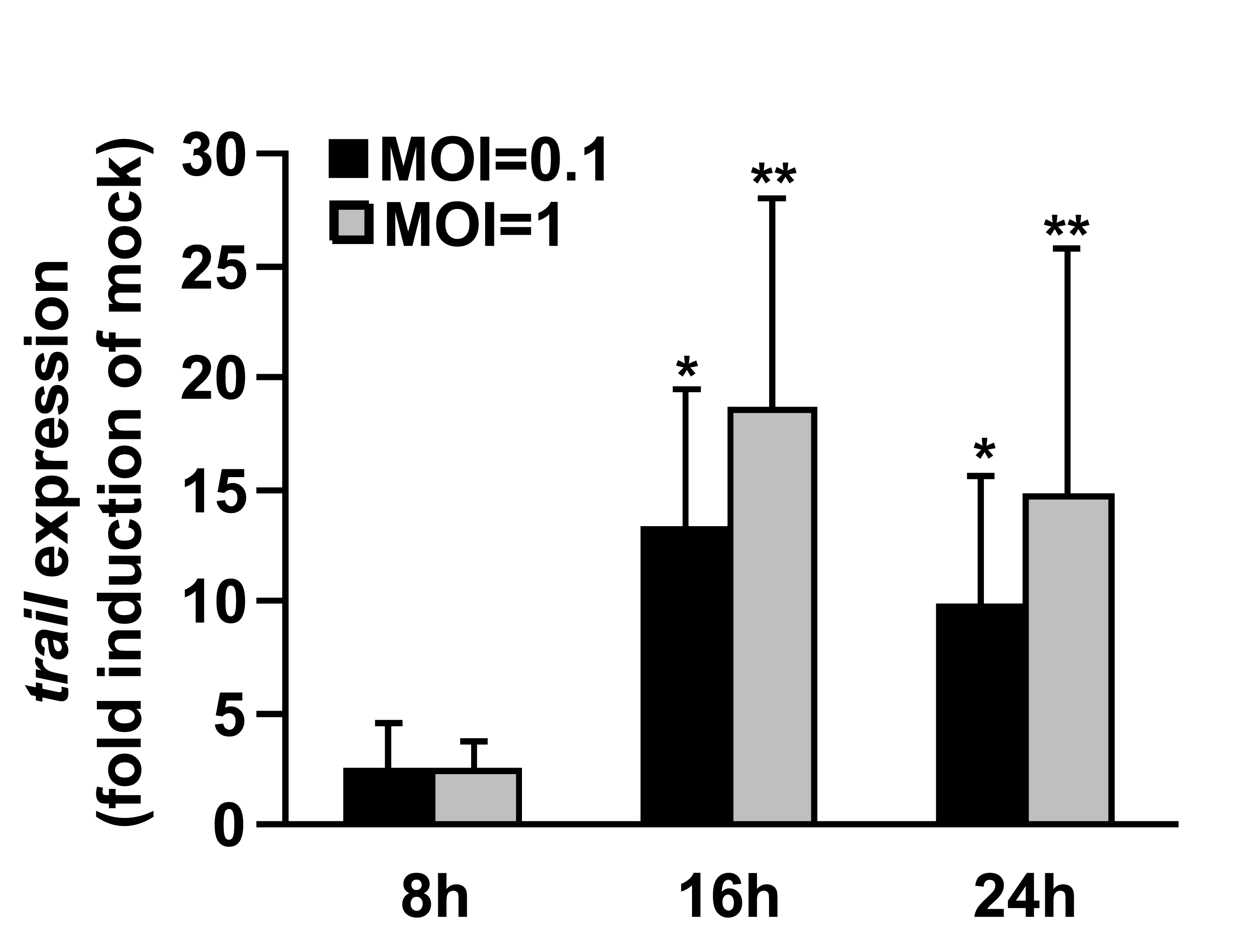

Supplement: Figure S4 — Time course of TRAIL expression in IV-infected AEC. Murine AEC were ex vivo infected with A/PR8 at the indicated MOI and TRAIL mRNA expression was quantified at the given times and is depicted as fold induction of mock-infected controls. Bar graphs represent means ± SD of 3 independent experiments. * p<0.05; ** p<0.01; ***p<0.001; MOI, multiplicity of infection; AEC, alveolar epithelial cells. (TIF) [file ppat.1003188.s004.tif]

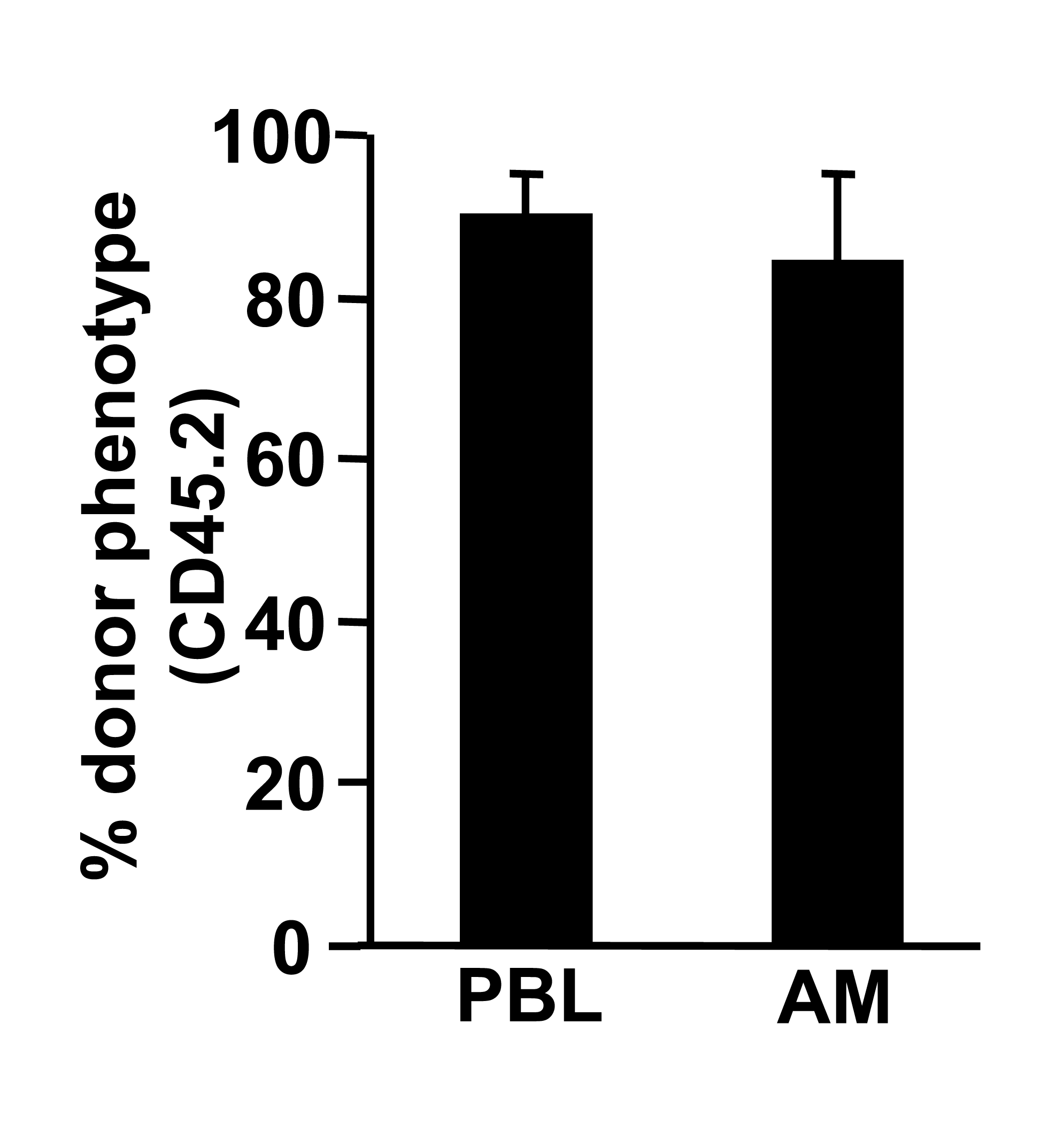

Supplement: Figure S5 — Efficiency of BM reconstitution and exchange of resident AM in chimeric mice. To control transplantation and resident AM reconstitution efficiency in CD45.1+ recipient mice after BMT with CD45.2+ BM, the fractions of CD45.2+ of total peripheral blood leukocytes (PBL) and of resident AM were quantified by flow cytometry from blood or BALF at 12w post BMT. Bar graphs show means ± SD from 3 independent experiments. (TIF) [file ppat.1003188.s005.tif]

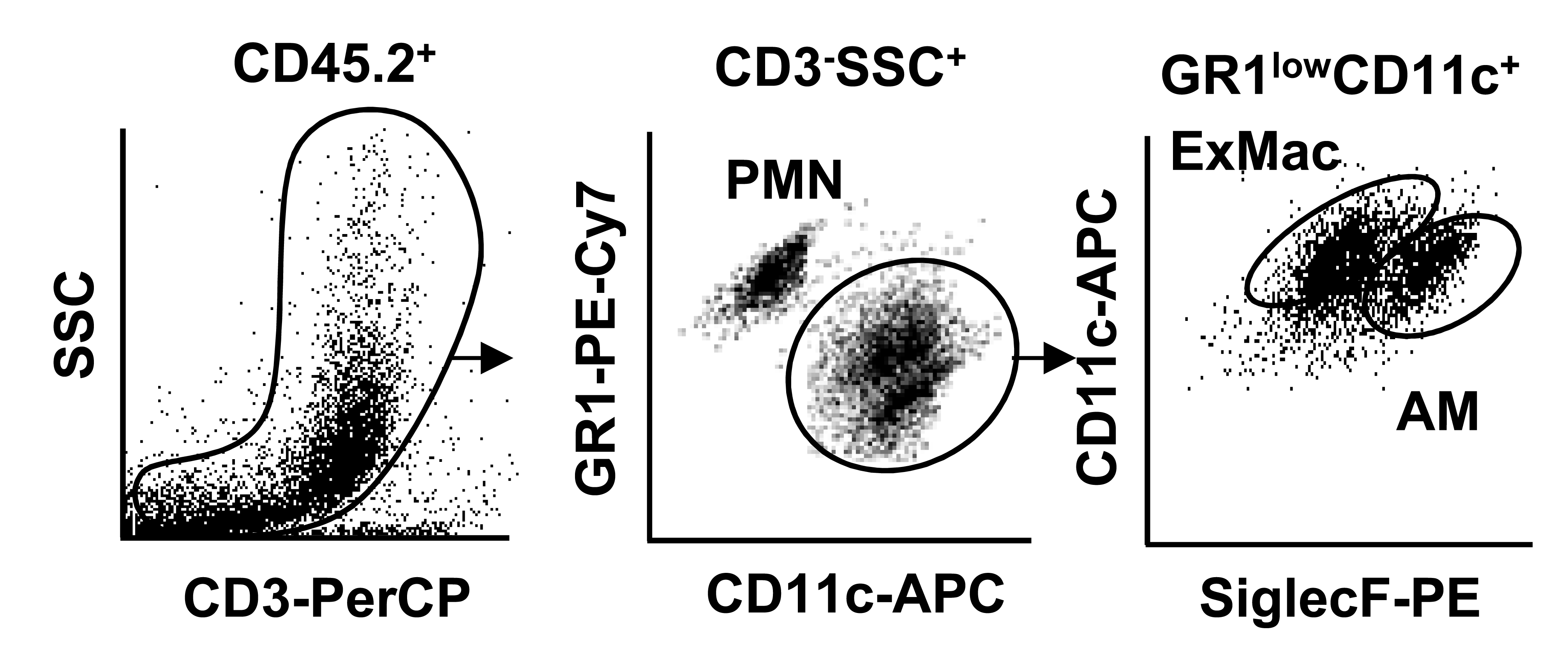

Supplement: Figure S6 — Gating strategies for detection of resident AM and exudate macrophages (ExMac) from BALF. Donor leukocytes (CD45.2+) were gated on the SSChighCD3ε− fraction to exclude lymphocytes. Neutrophils were defined as CD11c−GR-1high (PMN). ExMac were defined as CD11chighSiglecFlowGR-1int whereas resident AM were defined as CD11chighSiglecFhigh GR-1low. (TIFF) [file ppat.1003188.s006.tif]

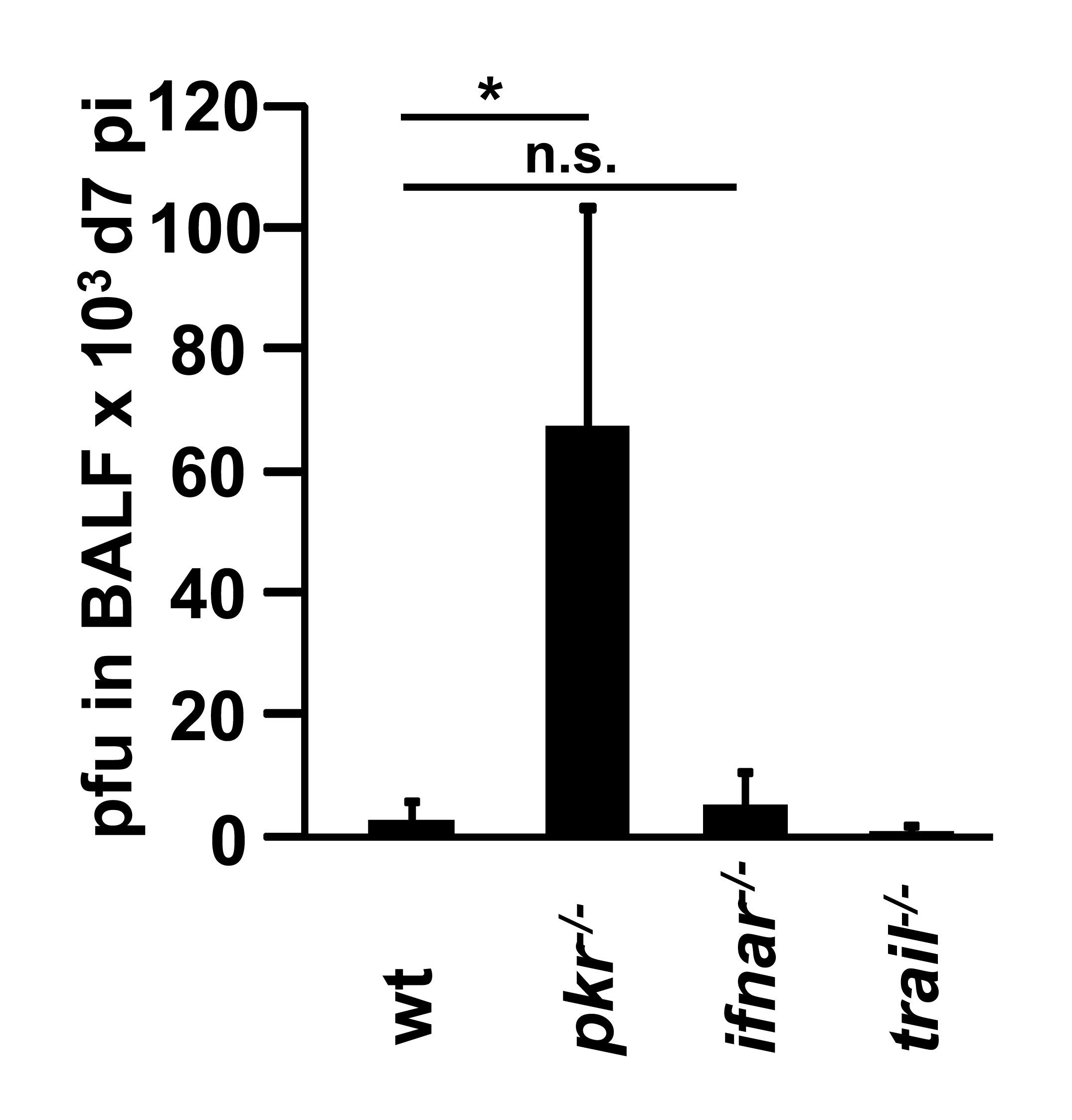

Supplement: Figure S7 — Viral loads in BALF of chimeric wt mice after transplantation of wt, pkr−/− , ifnar−/− or trail−/− BM at d7 pi. Bar graphs show means of pfu (plaque forming units)×103 ± SD of 5 animals/group. n.s., not significant. (TIFF) [file ppat.1003188.s007.tif]
